# Healthcare costs associated with gender dysphoria in children, adolescents and young adults in germany: A prevalence-based analysis using statutory health insurance data

**DOI:** 10.1007/s10198-025-01832-0

**Published:** 2025-08-20

**Authors:** Sophie Gottschalk, Claudia Konnopka, Katja Nettermann, Alicia Başoğlu, Ursula Marschall, Dirk Horenkamp-Sonntag, Angela Rölver, André Karch, Georg Romer, Hans-Helmut König

**Affiliations:** 1 Department of Health Economics and Health Services Research, University Medical Center Hamburg-Eppendorf, Hamburg Center for Health Economics, Martinistraße 52, Hamburg, 20246 Germany; 2 Institute of Epidemiology and Social Medicine, University of Münster, Münster, Germany; 3 Barmer, Wuppertal, Germany; 4 Techniker Krankenkasse, Healthcare Management, Hamburg, Germany; 5 Department of Child & Adolescent Psychiatry, Psychosomatics and Psychotherapy, University Hospital Münster, Münster, Germany

**Keywords:** Gender dysphoria, Gender incongruence, Healthcare costs, Health insurance, Germany

## Abstract

**Background:**

In the past decade, there has been an increase in individuals presenting to healthcare services with gender dysphoria (GD), the psychological distress that may arise when an individual’s birth-assigned sex does not align with his/her experienced gender. The current study aimed to analyze resource use and costs associated with prevalent GD in individuals aged 4 to 30 years.

**Methods:**

The analysis was a prevalence-based cost study using data of the two largest German health insurance funds (BARMER and TK) from 2018, 2019, and 2020. Individuals with prevalent GD were identified based on ICD-10 diagnosis codes related to gender dysphoria. These were compared to a control group balanced for (1) age, birth-assigned sex, degree of urbanization, and (2) additionally for psychiatric diagnoses using entropy balancing. Outcomes of interest were total and sector-specific annual costs (outpatient, inpatient, medications) and health-related resource use (hospital days, defined daily doses of medications). Groups were compared stratified by age groups, birth-assigned sex, and for a subgroup of individuals with GD receiving hormonal therapy.

**Results:**

Individuals with prevalent GD aged 4–30 years had higher average resource use and costs compared to controls, with little variation between years (e.g. difference in 2019 +€4,843 [95% confidence interval €4,306; €5,380], balanced for age, birth-assigned sex, degree of urbanization). The group difference was observed across age groups and healthcare sectors, with the largest differences found in somatic and psychiatric inpatient hospitalizations, and with psychiatric costs accounting for 50% of the total cost difference. Comparing individuals with GD receiving hormonal therapy with controls, the difference in total costs was similar, but the contribution of psychiatric costs was less pronounced (29%). The cost difference decreased considerably in all subgroups and sectors when psychiatric diagnoses were additionally balanced for.

**Conclusions:**

Individuals with GD aged 4–30 years had higher annual resource use and costs than controls. Future studies analyzing resource use and costs over multiple years and examining the temporal association between GD and psychiatric disorders would allow a more accurate estimate of the costs directly attributable to GD.

**Supplementary Information:**

The online version contains supplementary material available at 10.1007/s10198-025-01832-0.

## Background

According to the World Health Organization’s definition in the 11th revision of the International Statistical Classification of Diseases and Related Health Problems (ICD-11), individuals with gender incongruence (GI) experience a “marked and persistent incongruence between an individual´s experienced gender and the assigned sex, which often leads to a desire to ‘transition’, in order to live and be accepted as a person of the experienced gender, through hormonal treatment, surgery or other health care services to make the individual´s body align, as much as desired and to the extent possible, with the experienced gender” [1]. GI per se is not pathological (i.e. not a disease or disorder). This depathologization is reflected in the ICD-11, which classifies GI under “conditions related to sexual health” (HA6X), in contrast to its predecessor, the ICD-10, where GI is reflected in the subchapter “gender identity disorders” (F64.X) of the chapter F60-F69 “disorders of adult personality and behaviour”. However, GI can be associated with psychological distress/suffering, i.e. gender dysphoria (GD). The term GD was introduced in the 5th edition of the Diagnostic and Statistical Manual of Mental Disorders (DSM-5), emphasizing profound distress and suffering arising from the gender incongruence. GD is often used as a descriptive term without fulfilling the criteria for the psychiatric diagnosis.

Estimating the prevalence of individuals with GI/GD is challenging due to a lack of population-based studies and the use of different definitions and tools to identify “cases”, but in recent years, a substantial increase in individuals with GI/GD (children, adolescents, and adults) presenting to healthcare services could be observed [2–4].

Individuals with GD represent a vulnerable group with markedly higher rates of concurrent mental health diagnoses (e.g. depressive disorder, anxiety disorders, and attention deficit disorders, substance use/abuse disorders) compared to individuals without GD, although the prevalence rates of concurrent mental health diagnoses vary greatly between studies [5, 6]. In addition, adolescents and young adults with GD are more likely to exhibit suicidal ideation, life-threatening behavior, self-injurious thoughts or self-harm compared to cisgender peers [7]. These associations are usually explained by the marked body-related gender dysphoric distress [8, 9], as well as with the experience of stigma and discrimination-related stress under the minority stress model [10]. Beyond, there appears to be a slight diagnostic overlap between GD/GI and autism spectrum disorders, although its precise magnitude and possible underlying mechanisms are yet to be investigated [11]. Accordingly, and given the non-pathological nature of GI, treatment for GD does not target gender identity, but rather the mental, social and physical challenges/distress associated with a misalignment between birth-assigned sex and gender identity. Treatment options for gender-affirming medical interventions comprise psychological support and somatic, body-modifying interventions, i.e. hormonal therapy, phoniatric interventions, dermatological interventions, and gender-affirming surgery [12]. Especially for children and adolescents, there is controversy over treatment guidelines/recommendations, e.g. regarding the administration of puberty blockers to temporarily interrupt the progressing physical maturation during puberty before final decisions concerning irreversible body-modifying medical interventions are made [13–16].

In terms of treatment effectiveness, gender-affirming hormone therapy appears to reduce depressive symptoms and psychological distress, to improve quality of life and interpersonal functioning, and discontinuation rates of gender-affirming hormone therapy seem to be low [17, 18]. Similarly, gender-affirming surgery may increase life satisfaction, happiness and quality of life and reduce suicide attempts, self-harming, symptoms of gender dysphoria, anxiety and depression [19], although the effects were not always durable and individuals often still experience minority stress that impacts their mental health and quality of life [19]. Moreover, there is an indication of psychological interventions leading to improvement in various mental health outcomes [20]. However, overall, the quality of evidence for gender-affirming treatments still appears to be low, especially regarding the effects of puberty suppression and gender-affirming treatments in adolescents [21].

Specific challenges in the treatment of GD in children and adolescents arise from balancing the (parental) duty of care (§ 1626 German Civil Code) or protection from harm against the right to self-determination/personal freedom (Article 2 of the German Basic Law) as well as the question of the minor’s ability to make informed decisions. Ravindranath et al. concluded that adolescents have the neurocognitive developmental ability to make such informed decisions when the decision-making process takes place over a sufficiently long period of time, is based on informed discussion and a supportive environment, rather than being “rushed”, where immediate rewards, affective processes, and peers may overly influence the decision [16, 22, 23].

Unlike in other countries, statutory health insurance in Germany comprehensively covers the costs of gender-affirming medical treatment (BSG 3 RK 15/86), but there are a number of barriers and criteria in place. E.g., the reimbursement of gender-affirming surgery is tied to the ICD-10 diagnosis of “transsexualism” (F64.0) and is only granted after several months of psychotherapy have not solved the “problem” [24, 25]. These criteria are contrary to the recommendations of clinical or professional associations [26].

Irrespective of the current discussions on the adequate treatment of individuals with GD, including children and adolescents, this group is increasingly making use of healthcare services. However, little is known about the quantity and pattern of this resource use in Germany and internationally. Given the increase in prevalence of health services utilization by this population, describing and monitoring of resource use and the associated costs is becoming more relevant. Such analyses could, e.g., aid health insurances with planning budgets or identifying areas of optimizing care pathways.

For the US setting and based on a sample of privately insured people, Baker and Restar found trends of increasing use of gender-affirming hormone therapy and surgery and associated increase in total costs between 2011 and 2019 [27]. They concluded that, despite this increase, the impact on the payer’s budget remained minimal. The trend of increasing gender-affirming surgeries and associated costs in the US is supported by Chu et al. who analyzed a representative pool of inpatient visits [28]. For Germany, Grochtdreis et al. analyzed the excess costs of transgender and gender diverse individuals with GI/GD compared to a general population control group [29]. The authors observed on average higher costs of the GI/GD group, mainly attributable to higher indirect costs due to absenteeism from work, but this difference was not statistically significant. However, the generalizability of these results is limited as the analysis was based on a selected population of individuals with GI/GD eligible for a randomized-controlled trial that included participants aged 18 years and older from remote areas in northern Germany who had not yet started transition-related treatments.

The TRANSKIDS-CARE project used claims data from two large statutory health insurance funds to describe the care situation for children, adolescents, and young adults with GD in Germany on an epidemiological and health economic basis. This paper is part of the health economic analyses within the project and reports on a prevalence-based cost study. Specifically, the current study aimed to analyze resource use and healthcare costs associated with prevalent GD in individuals aged 4 to 30 years in the years 2018, 2019, and 2020. This included identifying the healthcare sectors in which costs were incurred, describing potential changes over the time, and investigating the role of hormonal therapy.

## Methods

### Study design and sample

This study was a prevalence-based cost analysis from a healthcare payer’s perspective, based on individual-level claims data (bottom-up approach) for the years 2018, 2019, and 2020 from the two largest German statutory health insurance funds (BARMER and Techniker Krankenkasse [TK]) which together cover about 24% of the German population. In Germany, health insurance is mandatory and almost 90% of the population are insured by the statutory health insurance scheme, which is financed by income-related contributions. Individuals are free to choose their insurance provider from more than 90 statutory health insurance funds. These funds have a common benefits catalog covering most healthcare services (i.e., inpatient and outpatient services, medications).

The population of interest in this study were individuals aged 4 to 30 years with prevalent GD in the respective year. These were compared to a control group which was pre-selected by the insurance funds, consisting of individuals in the claims data who never had a GD diagnosis between 2017 and 2020 but were of similar age, sex, and area of residence as the population with prevalent GD.

### Identification of cases

Prevalent cases of GD in the respective year (2018, 2019, 2020) were identified based on recorded ICD-10 diagnosis codes F64 (Gender identity disorders) and F66 (Psychological and behavioral disorders associated with sexual development and orientation). Cases with code F64.1 (Dual-role transvestism) were excluded, as well as cases with code F66.2 (Sexual relationship disorder), if there was no concurrent F64 diagnosis. Furthermore, individuals with diagnoses related to differences of sex development (DSD) were excluded. In line with recommendations for the analysis of German health insurance data, diagnoses in the outpatient sector had to be coded as confirmed/secured (i.e. no suspected diagnoses; Table S1) and documented in at least two quarters in the respective year (M2Q criterion) to be counted as prevalent cases; in the inpatient sector, primary and secondary diagnoses were considered (no M2Q criterion) [30]. Please note that excluded cases were excluded from the analysis and not assigned to the control group.

For subgroup analyses, cases with GD and receiving hormonal therapy (gender-affirming hormones or puberty blockers) were identified based on recorded medications with the following Anatomical-Therapeutic-Chemical (ATC) codes: G03BA03 (testosterone), G03CA01, G03CA03 and G03CA04 (estrogen), H01CA04 and H01CA07 (gonadotropin-releasing hormone), and L02AE02 and L02AE04 (gonadotropin-releasing hormone analogues).

The administrative sex of individuals with GD in the insurance data may differ from their birth-assigned sex (e.g. if the registered sex has already been changed), which was reconstructed when necessary. Birth-assigned sex was recoded as “female” if the individual had ever received testosterone (ATC G03BA02) or undergone female gender-affirming surgery (procedure codes [OPS] 5-646.0, 5-643.2, 5-683). It was recoded as “male” if the individual had ever received estrogen (ATC G03CA01, G03CA03, G03CA04) or undergone male gender-affirming surgery (OPS codes 5-646.1, 5-705.6).

### Outcomes – Assessment of resource use and costs

Outcomes of interest in this study were total and sector-specific annual costs from the healthcare payer’s perspective as well as health-related resource use, both drawn from claims data of the BARMER and TK health insurance funds from the years 2018, 2019, and 2020. Healthcare sectors considered were outpatient physician services (psychiatric [including psychiatrists and psychotherapists], general practitioner, other), outpatient hospital treatments (psychiatric, somatic), inpatient hospitalizations (psychiatric, somatic), and medications (psychiatric, somatic). In terms of resource consumption, the days of psychiatric and somatic hospitalization and the defined daily doses (DDD) of psychiatric and somatic medication were considered. To ensure comparability between years, costs occurring in the years 2018 and 2019 were inflated to 2020 euros (€) based on the consumer price index [31].

### Statistical analysis

Costs and resource use in each year were compared between groups (individuals with prevalent GD vs. controls; individuals with GD and receiving hormonal therapy vs. controls). To achieve comparability between groups, entropy balancing was performed, i.e., data were pre-processed by re-weighting sample units of the control group to match the GD group on mean, variance and skewness of selected covariates, thereby achieving covariate balance [32]. In a first step, groups were balanced for age (continuous), birth-assigned sex (male/female), degree of urbanization (administratively independent city, urban, rural, sparsely populated) (Tables S2 & S4). In a second step, groups were additionally balanced for selected psychiatric diagnoses (recorded in the respective year) to investigate their potential confounding effect on the results, assuming that psychiatric diagnoses do not only occur parallel to and as a consequence of GD, but might precede GD and represent a risk factor/determinant for the development of GD [5] (Tables S3 & S5). This re-weighting procedure was performed separately for each year and group comparison (GD vs. control and GD receiving hormonal therapy vs. control). The weights were then applied to subsequent analyses. The mean of costs or resource use variables was calculated for each group. Mean between-group differences were calculated based on linear regression models and reported alongside the corresponding 95% confidence intervals (CI) derived from bootstrapped standard errors (1,000 replications). Furthermore, group comparisons were stratified by age group (4–12, 13–17, 18–24, 25–30 years) and birth-assigned sex (female, male).

All analyses were conducted using STATA/MP 18.0 [StataCorp. 2023. Stata Statistical Software: Release 18. College Station, TX: StataCorp LLC].

## Results

Among individuals aged 4 to 30 years insured with the TK and BARMER health insurance funds, *n* = 2,837 (2018), *n* = 3,479 (2019), and *n* = 4,086 (2020) individuals with prevalent GD were identified. Of these, more than half also received hormonal therapy in the respective year (i.e. puberty suppressing or gender-affirming medications that are administered earliest after the onset of puberty): *n* = 1,579 (56%) in 2018, *n* = 2,031 (58%) in 2019, and *n* = 2,567 (63%) in 2020.

### Individuals with prevalent GD vs. control

Compared to the pre-selected control group and before balancing, the share of birth-assigned females was lower in individuals with prevalent GD (66% vs. 72% in 2019), and the share of individuals with psychiatric diagnoses in the same year was higher (e.g. in 2019, 44% vs. 9% were diagnosed with affective disorder [F30-F39], 27% vs. 8% with reaction to severe stress and adjustment disorders [F43], and 14% vs. 7% with somatoform disorders [F45]) (Tables S2-S5). After entropy balancing, groups were comparable in the prespecified variables.

**Fig. 1:**
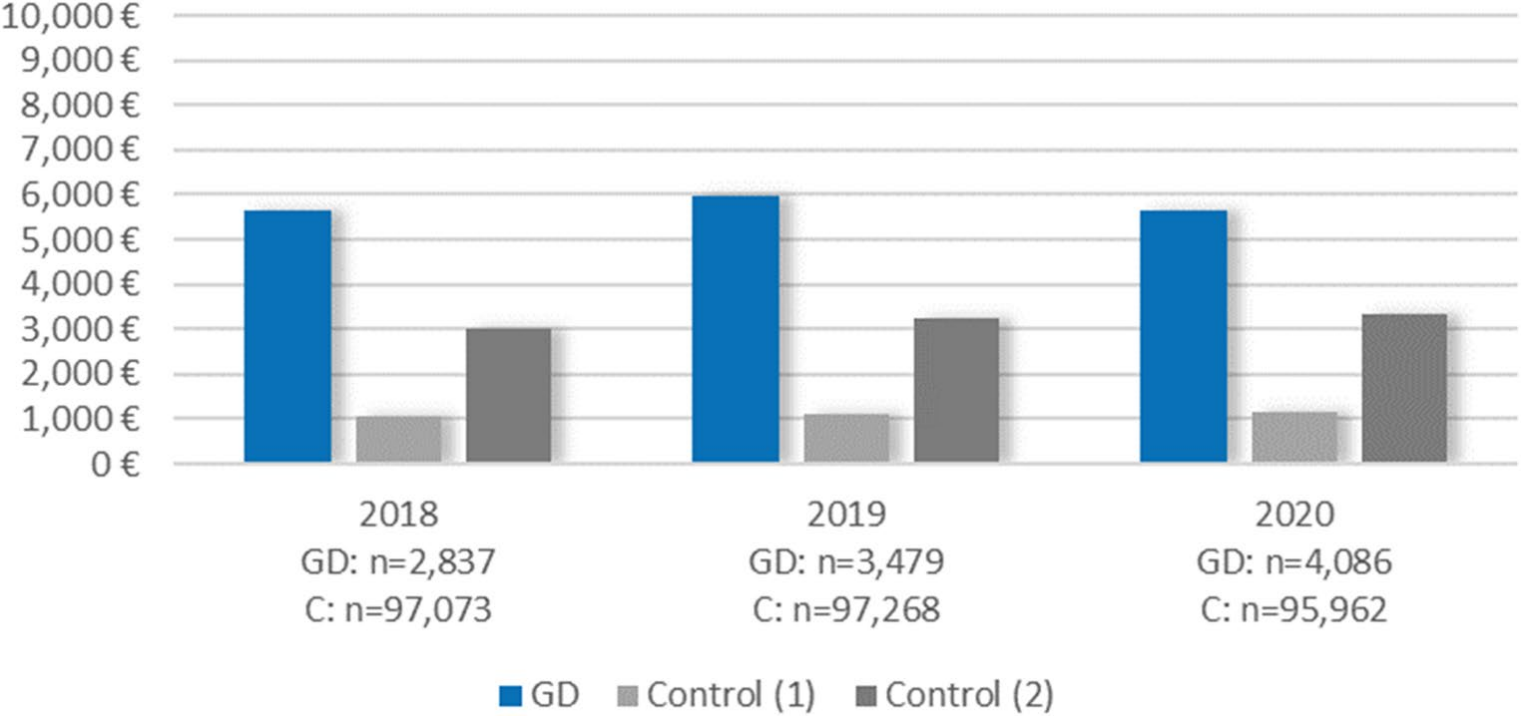
Mean total costs for individuals with prevalent GD vs. controls by year. Control (1) = balanced for age, birth-assigned sex, and degree of urbanization; Control (2) = balanced for age, birth-assigned sex, degree of urbanization, and psychiatric diagnoses

Balancing for age, birth-assigned sex and degree of urbanization, individuals with prevalent GD had higher average total costs than the control group in all observed years (2018, 2019, 2020), with a cost difference of e.g. €4,843 (95% CI €4,306 to €5,380) in 2019 (Fig. 1; Table 1). No relevant difference was observed between years; the pattern looked similar with average total costs in the GD group between €5,500 and €6,000 and around €1,000 in the control group.

**Table 1:** Differences in costs in € (total and by healthcare sector) and resource use between individuals with prevalent GD in 2019 vs. controls alongside 95% confidence intervals

	GD (*n* = 3,479) vs. control (*n* = 97,268)		GD with hormonal therapy (*n* = 2,031) vs. control (*n* = 97,268)	
	(1)	(2)	(1)	(2)
Costs				
Total	4,843 (4,306; 5,380)	2,753 (2,163; 3,343)	5,181 (4,391; 5,972)	3,600 (2,796; 4,405)
By healthcare sector				
Outpatient	988 (942; 1,034)	545 (495; 596)	978 (920; 1,036)	588 (526; 649)
GP	105 (98; 111)	56 (49; 63)	111 (103; 119)	67 (59; 75)
Psychiatric	699 (657; 741)	355 (309; 401)	611 (561; 662)	311 (257; 365)
Other	184 (169; 200)	135 (118; 151)	255 (233; 277)	210 (187; 232)
Hospital inpatient	3,122 (2,608; 3,635)	1,714 (1,152; 2,275)	3,351 (2,581; 4,120)	2,367 (1,586; 3,147)
Psychiatric	1,517 (1,248; 1,785)	291 (−52; 634)	736 (520; 952)	−83 (−326; 159)
Somatic	1,605 (1,164; 2,046)	1,423 (979; 1,867)	2,614 (1,872; 3,357)	2,450 (1,705; 3,194)
Hospital outpatient	264 (239; 290)	152 (124; 180)	232 (201; 263)	145 (113; 177)
Psychiatric	147 (129; 166)	91 (71; 111)	108 (86; 131)	67 (45; 90)
Somatic	117 (100; 134)	61 (42; 81)	124 (103; 145)	78 (56; 100)
Medication	469 (343; 596)	342 (208; 476)	621 (493; 749)	501 (365; 637)
Psychiatric	41 (34; 48)	4 (−5; 14)	33 (26; 39)	2 (−6; 10)
Somatic	428 (303; 554)	338 (205; 471)	588 (461; 716)	499 (364; 635)
Resource use				
Days in hospital	6.2 (5.4; 7.0)	2.2 (1.2; 3.1)	5.4 (4.5; 6.2)	2.2 (1.3; 3.1)
Psychiatric	4.3 (3.6; 5.0)	0.7 (−0.2; 1.5)	2.4 (1.7; 3.1)	−0.4 (−1.2; 0.4)
Somatic	1.9 (1.6; 2.2)	1.5 (1.2; 1.9)	2.9 (2.4; 3.4)	2.6 (2.2; 3.1)
DDD	342 (326; 358)	256 (238; 273)	502 (480; 523)	419 (397; 442)
Psychiatric	61 (54; 67)	9 (1; 17)	59 (50; 67)	9 (−1; 18)
Somatic	281 (267; 295)	246 (231; 262)	443 (424; 462)	411 (392; 430)
(1) = Balanced for age, birth-assigned sex, county type; (2) = Balanced for age, birth-assigned sex, county type, and psychiatric diagnoses.				

**Fig. 2:**
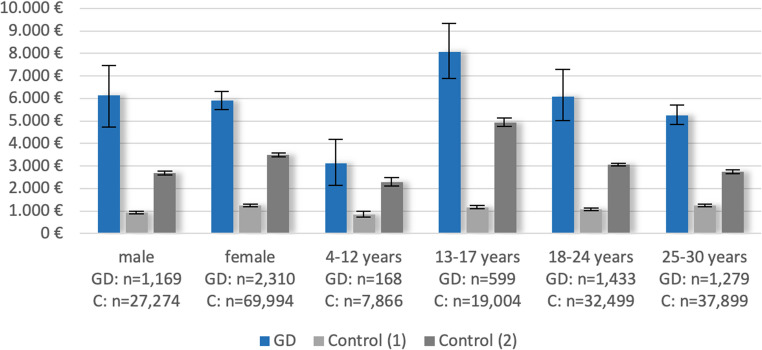
Mean total costs 2019 (with 95% confidence intervals) for individuals with prevalent GD vs. controls by birth-assigned sex and age groups. Control (1) = balanced for age, birth-assigned sex, and degree of urbanization; Control (2) = balanced for age, birth-assigned sex, degree of urbanization, and psychiatric diagnoses

Higher average total costs of individuals with prevalent GD compared to controls were observed in 2019 in birth-assigned male and female sex as well as across age groups (Fig. 2), with the costs of GD cases being highest in the age group 13–17 years (€8,076), resulting in the cost difference also being highest in this age group.

**Fig. 3:**
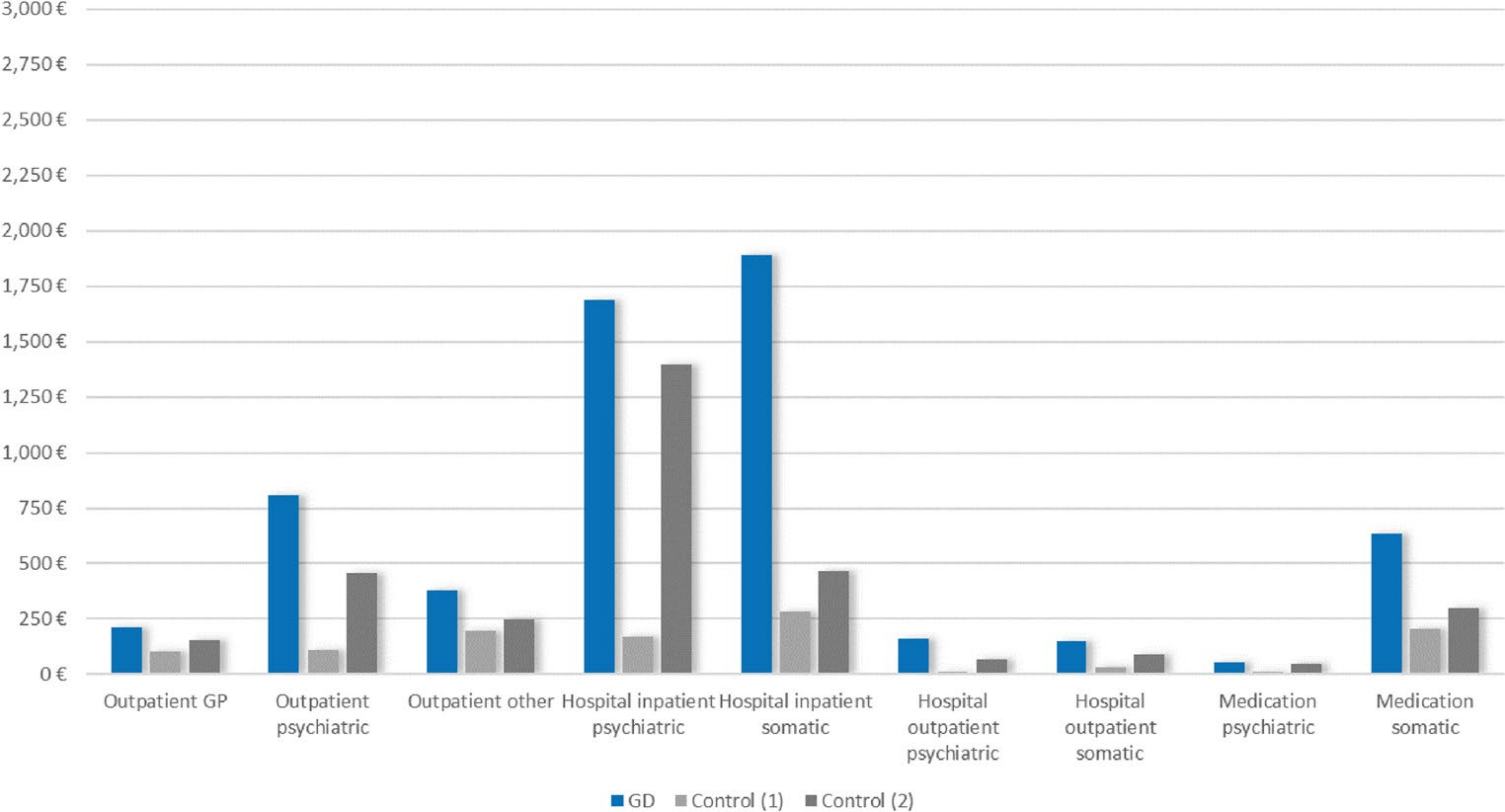
Mean costs 2019 for individuals with prevalent GD (*n* = 3,479) vs. controls (*n* = 97,268) by healthcare sectors. Control (1) = balanced for age, birth-assigned sex, and degree of urbanization; Control (2) = balanced for age, birth-assigned sex, degree of urbanization, and psychiatric diagnoses

Individuals with prevalent GD had higher average costs (2019) than controls across all healthcare sectors (Fig. 3; Table 1), the largest between-group differences being observed in somatic inpatient hospitalization (+€1,605, 95% CI €1,164 to €2,046, accounting for 33% of the total cost difference between groups), followed by psychiatric inpatient hospitalization (+€1,517, 95% CI €1,248 to €1,785, ≙ 31%), outpatient psychiatric service use (+€699, 95% CI €657 to €741, ≙ 14%), and somatic medication use (+€428, 95% CI €303 to €554, ≙ 9%). Furthermore, individuals with prevalent GD spent on average more days in psychiatric (+ 4.3, 95% CI 3.6 to 5.0) and somatic hospitals (+ 1.9, 95% CI 1.6 to 2.2) in 2019 compared to controls (Table 1, Table S6). Similarly, their medication use was higher, with an average difference of + 61 DDD (95% CI 54 to 67) for psychiatric and + 281 DDD (95% CI 267 to 295) for somatic medications. The differences in costs and resource use corresponded to a higher proportion of users in the GD group compared to the control group in all categories (Table S6).

Additionally balancing the groups for psychiatric diagnoses substantially reduced the between-group difference in costs and resource use across all subgroups and sectors (Figs. 1, 2 and 3; Table 1, Table S6). For example, the difference in average total costs 2019 was reduced by 43% to +€2,753 (95% CI €2,163 to €3,343). This reduction was mainly attributable to the reduced difference in costs for psychiatric inpatient hospitalizations (+€291, 95% CI -€52 to €634) and psychiatric outpatient services (+€355, 95% CI €309 to €401).

### Individuals with prevalent GD receiving hormonal therapies vs. control

**Fig. 4:**
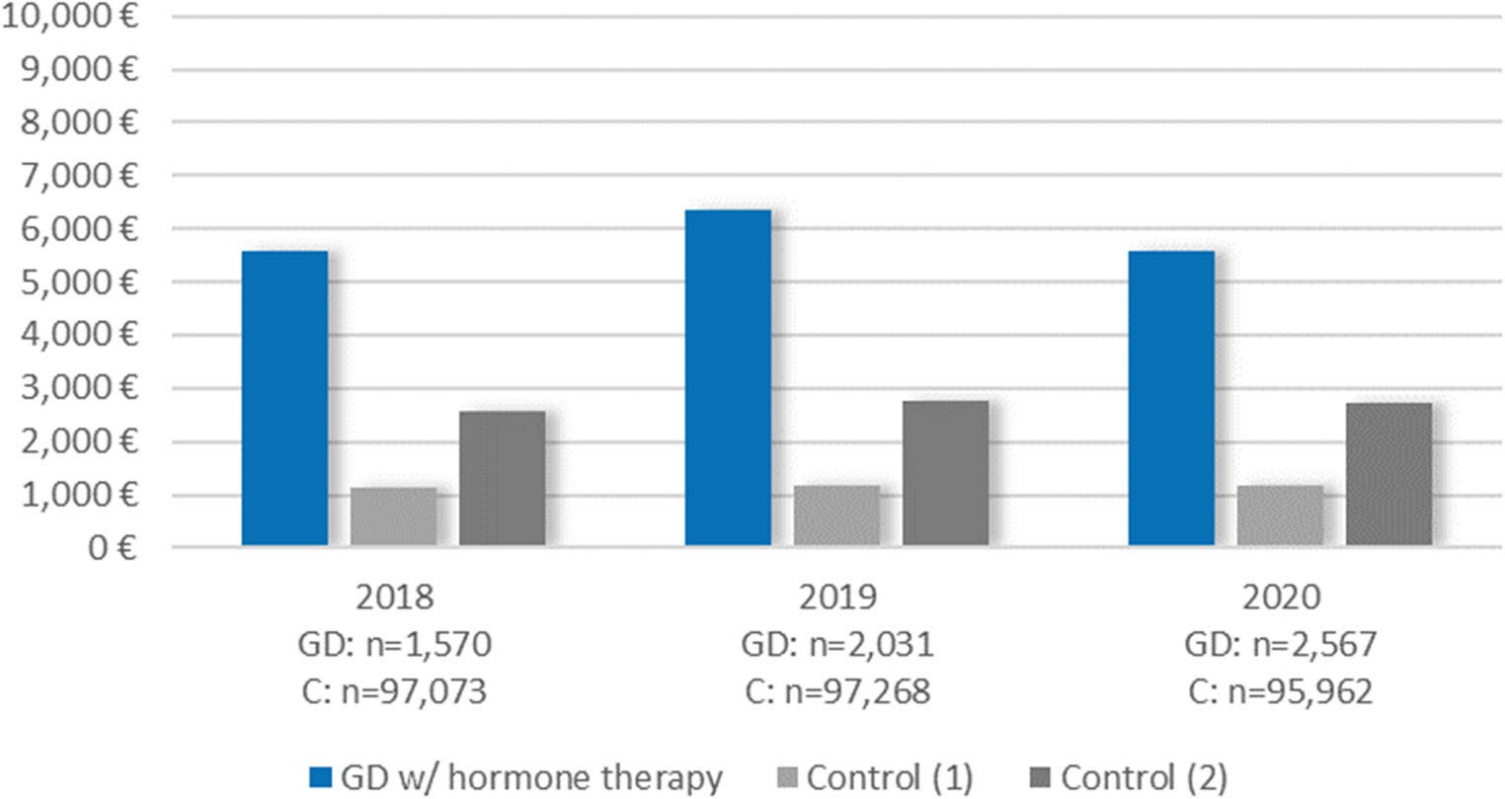
Mean total costs for individuals with prevalent GD and receiving hormonal therapy vs. controls by year. Control (1) = balanced for age, birth-assigned sex, and degree of urbanization; Control (2) = balanced for age, birth-assigned sex, degree of urbanization, and psychiatric diagnoses

**Fig. 5:**
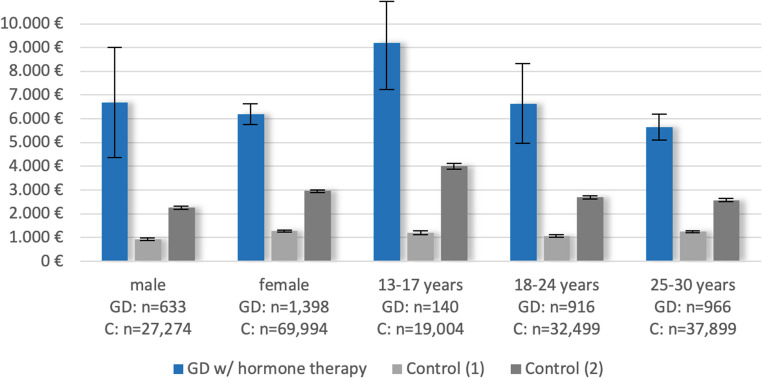
Mean total costs 2019 (with 95% confidence intervals) for individuals with prevalent GD and receiving hormonal therapy vs. controls by birth-assigned sex and age groups. Control (1) = balanced for age, birth-assigned sex, and degree of urbanization; Control (2) = balanced for age, birth-assigned sex, degree of urbanization, and psychiatric diagnoses

**Fig. 6:**
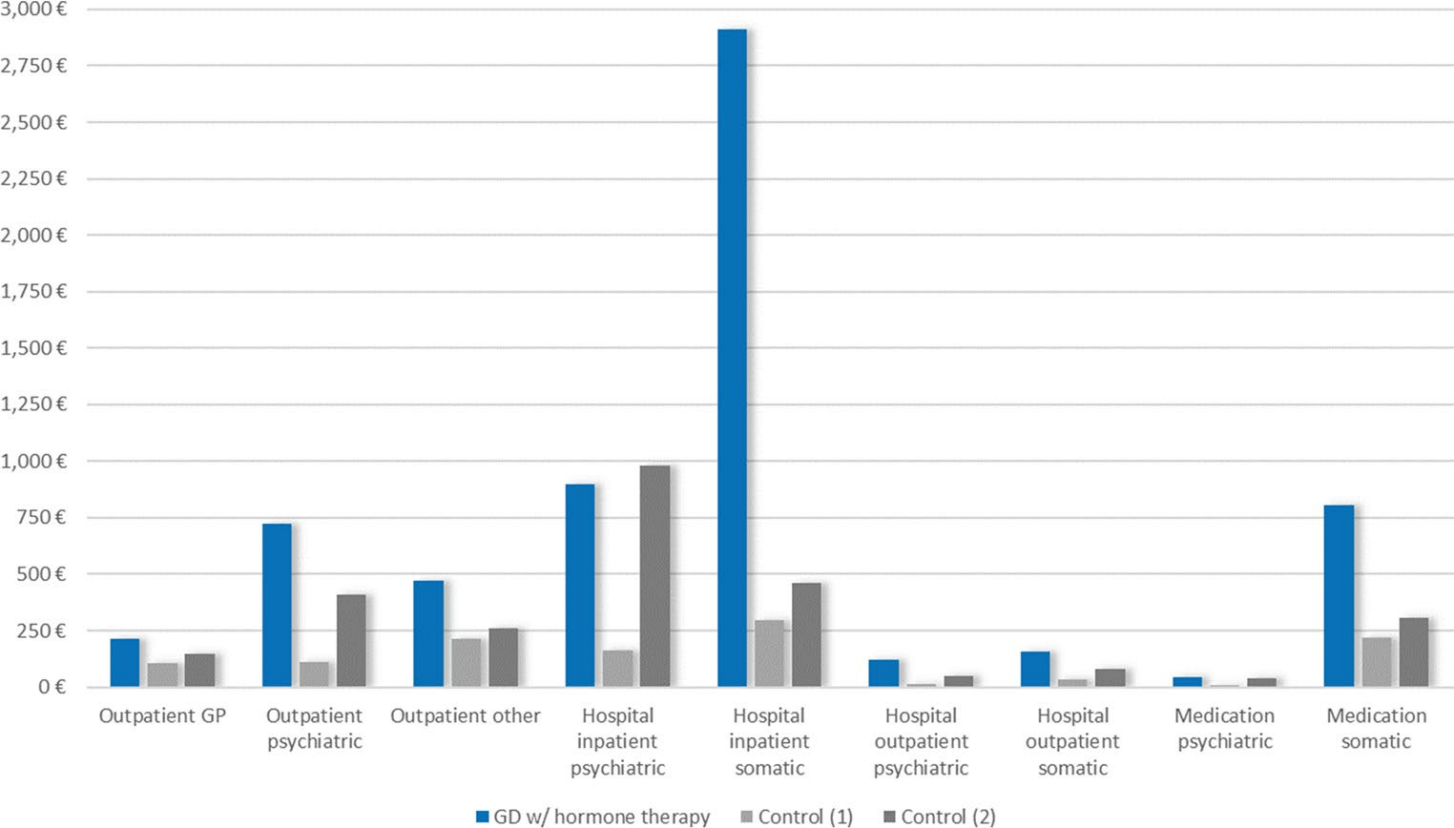
Mean costs 2019 for individuals with prevalent GD and receiving hormonal therapy (*n* = 2,031) vs. controls (*n* = 97,268) by healthcare sectors. Control (1) = balanced for age, birth-assigned sex, and degree of urbanization; Control (2) = balanced for age, birth-assigned sex, degree of urbanization, and psychiatric diagnoses

The results showed a similar picture when comparing individuals with prevalent GD receiving hormonal therapy with controls (Figs. 4, 5 and 6; Table 1, Table S7). Balancing groups for age, birth-assigned sex, and degree of urbanization, the difference in total costs in 2019 between prevalent GD cases receiving hormonal therapies and controls was slightly higher (+€5,181, 95% CI €4,391 to €5,972), and the difference in somatic inpatient hospitalization costs was especially pronounced (+€2,614, 95% CI €1,872 to €3,357). With additional balancing for psychiatric diagnoses, the cost differences again became smaller; for inpatient psychiatric hospitalizations, the difference was even eliminated/reversed (-€83, 95% CI -€326 to €159).

## Discussion

This study aimed to analyze resource use and costs associated with prevalent GD in individuals aged 4 to 30 years insured with the TK and BARMER statutory health insurance funds in 2018, 2019, and 2020. Individuals with GD had, on average, considerably higher resource use and healthcare costs compared to controls balanced for age, birth-assigned sex, and degree of urbanization, with average total costs in the GD group between €5,500 and €6,000 and around €1,000 in the control group. The group difference was observed across all healthcare sectors, showed little variation between years, and the difference in total costs was similar when the comparison was restricted to individuals with GD receiving hormonal therapy.

In 2019, the average total healthcare costs of individuals with GD exceeded those of the control group balanced for age, birth-assigned sex, and degree of urbanization by €4,843 (95% CI €4,306 to €5,380). 64% of this difference was attributable to inpatient hospitalizations (of which about half was attributable to psychiatric and somatic hospitalizations, respectively), 20% to the outpatient sector (of which 70% was attributable to psychiatric services), and 10% to medication (of which 90% was attributable to somatic medication). Overall, 50% of the total cost difference was due to psychiatric care. It should also be noted that while inpatient psychiatric and somatic hospitalizations contributed most to the cost difference, only a minority of individuals with prevalent GD had any inpatient hospitalization (9% psychiatric and 24% somatic in 2019). In contrast, a majority received outpatient psychiatric treatment: 61% in the total sample vs. 10% (33% when also balanced for psychiatric diagnoses) in the control group, with an especially high proportion of users in the age group 13–17 years (70%). This may reflect current treatment patterns including diagnostic mental health assessments and monitoring before and after starting hormonal treatments, the requirement of several months of psychotherapy to qualify for reimbursement of medical gender-affirming surgery [24, 25] as well as psychotherapy needs for concurrent mental health problems.

In 2019, 58% of individuals with GD also received hormonal therapies (gender-affirming hormones or puberty blockers). Although the difference in total costs was similar when comparing this GD subgroup to controls (+€5,181, 95% CI €4,391 to €5,972), the shares of the healthcare sectors in the total cost difference varied: Psychiatric costs accounted for only 29% of the cost difference, whereas somatic costs played a larger role, especially cost for somatic inpatient hospitalizations, which alone accounted for 50% of the total cost difference. Also, the proportion of individuals contributing to the average somatic inpatient costs was somewhat higher (32%) than in the total group of individuals with GD. This could reflect a gender-affirming treatment path, i.e. individuals who have started taking gender-affirming hormones may be more likely to proceed to surgical body-modifying interventions, which comes along with higher somatic hospitalization costs.

The observation of individuals with GD having higher average total healthcare costs than controls held true across all age groups, but this cost difference was less pronounced in children aged 4–12 years. This is likely to reflect that before the onset of puberty there is no rationale nor treatment recommendation for any kind of somatic interventions in gender dysphoria.

Additionally balancing the groups for prevalent psychiatric diagnoses considerably reduced the cost difference (e.g., the difference in inpatient psychiatric hospitalization costs was no longer significant). This may suggest that a large part of the cost difference between groups is due to the treatment of concurrent psychiatric conditions, which are more prevalent in individuals with GD than in controls. This increased prevalence is frequently explained with gender dysphoric distress related to bodily sex characteristics [8, 9] as well as experienced minority stress of individuals with GI/GD [10]. Thus, controlling for psychiatric conditions would potentially underestimate the true costs associated with GD, as concurrent psychiatric conditions could be seen as partial mediators of the costs associated with GD. Taken further, this could imply that measures to reduce stigmatization/discrimination may help dampen the psychological distress and development of psychiatric disorders and consequently reduce costs. However, not accounting for concurrent psychiatric conditions at all could also overestimate the costs associated with GD, as the temporal-causal direction of the association between GD and psychiatric disorders is not entirely understood (e.g., it cannot be excluded that some individuals develop GD in reaction to preceding psychological stressors/conditions) [5]. Either way, individuals with GD showed higher costs overall than their controls, indicating that they have higher resource use irrespective of concurrent psychiatric diagnoses. It remains unclear whether this difference is explained by the direct treatment of GD alone or additionally by other factors not measured or included in the entropy balancing.

Based on these considerations, it is also relevant to further investigate the extent to which gender-affirming treatments have (long-term) positive effects on concurrent mental health outcomes in individuals with GI/GD, which may also influence healthcare costs in the long-term. Although a recent systematic review found gender-affirming hormone therapy to apparently reduce depressive symptoms and psychological distress, improve quality of life and interpersonal functioning, the authors also emphasized that the quality of evidence did not allow causal inference as randomized controlled trials are not applicable in this field, and outcome data usually are based on uncontrolled longitudinal cohort studies [17].

### Limitations and future research perspectives

Several limitations as well as future research perspectives that can be derived from the presented study should be highlighted.

The current study was based on claims data from two large statutory health insurance funds in Germany, meaning that the cost perspective was limited to that of the healthcare payer, and it cannot be ruled out that people from other health insurance funds may differ in certain characteristics, which could affect the utilization of healthcare resources and costs and thus lead to divergent results. Given differing reimbursement structures and (financial) access barriers to GD treatment (the latter being rather low in the statutory health insurance system in Germany), the transferability of the results to other countries or healthcare systems is limited.

Prevalent cases with GD were identified based on diagnoses according to the ICD-10 (which is still binding for reimbursement purposes in Germany), meaning that some individuals who would qualify as gender incongruent according to the new ICD-11 diagnosis code (HA6X) were potentially missed if they did not fulfill diagnostic criteria for ICD-10 diagnoses related to GD. In addition, the application of the recommended M2Q criterion to identify cases may have led to an underestimation of prevalent cases of individuals with GD [30]. Consequently, individuals with prevalent GD in this study are not representative of the overall population of individuals with GI/GD aged 4–30 years, with a possible bias towards cases with more health care needs and higher costs due to rather strict inclusion criteria.

Furthermore, the study only examined the annual resource use and costs of individuals with prevalent GD in the respective year, instead of analyzing the development of resource use and costs over several years (including the time before and after the first incidence of GD), which would, e.g., shed some light on the temporal association between the occurrence of GI/GD and mental health conditions. Moreover, although no relevant variation in the cost difference between groups could be observed between 2018 and 2020, an effect of the COVID-19 pandemic on the resource use and costs of individuals with and without GD cannot be excluded. For this reason, most of the results reported in this study were for 2019. Finally, the group of individuals with GD was very small compared to the control group, which could have limited the power of the analysis.

The study’s results indicated that the annual costs of treating individuals with GD (including psychotherapy, potential gender-affirming hormone therapy and surgery) and concurrent psychiatric disorders in individuals aged 4–30 years exceed the costs of the control group. However, NOT treating individuals with GD could also have long-term health and economic consequences. The minority stress potentially experienced by people with GD might trigger both behavioral (e.g. substance abuse) and physiological stress responses, which are associated with the development of stress-related health outcomes (e.g. metabolic and cardiovascular diseases) and could result in high healthcare costs in the long term [33, 34]. Furthermore, the consequences may extend beyond the healthcare system. For example, Grochtdreis et al. found higher rates of workplace absenteeism related to mental health problems and associated costs due to lost productivity in treatment-seeking individuals with GI/GD, even after controlling for somatic and psychiatric diagnoses [29]. This suggests that treating GD could potentially be cost-effective, when considering all potential long-term health and economic consequences and benefits.

Few studies to date have examined the cost-effectiveness of treatments related to GI/GD. For example, Padula et al. conducted a model-based analysis in the US setting and found insurance coverage for medically necessary transgender-related services to be cost-effective with an incremental cost-effectiveness ratio of $9,314 per quality-adjusted life year gained [35]. They also emphasized that the budget impact of covering medically necessary transgender-related services was marginal per member and month. Even if these results are hardly transferable to the German setting, the proportion of individuals with GD in the total insured population is also very low here. This means that the costs of treating all individuals with GD only account for a small proportion of the total health insurance expenditure. However, given the increasing prevalence and the controversial nature of the topic, it remains important to investigate and objectively report the long-term effects on health and costs of different domains in the treatment of individuals with GD in further studies.

## Conclusions

On average, individuals with prevalent GD had considerably higher resource use and costs compared to controls. The group difference was observed across all healthcare sectors and showed little variation between years. The difference in total costs was similar when the comparison was restricted to individuals with GD receiving hormonal therapies but the contribution of individual healthcare sectors to the total cost difference varied. Balancing the groups for prevalent psychiatric diagnoses considerably reduced the cost difference. Future studies analyzing resource use and costs over multiple years, examining the temporal-causal relationship between GD and psychiatric disorders as well as the long-term effects on gender-affirming treatments on health and costs, would allow a more accurate estimation of costs directly attributable to GD and GD-related treatment. The question, to which degree body-modifying medical interventions aiming at an improvement of body satisfaction and a reduction of gender dysphoric body-related distress are associated with a reduction of psychiatric health care costs as a possible indicator for a reduction of psychiatric morbidity, however, cannot be answered by our prevalence-based analyses. It is not possible to predict accurately how the prevalence of GD/GI and the use of healthcare services will develop over the next few years. However, on the basis of current prevalence trends, a further increase can be assumed, especially among adolescents and young adults.

## Supplementary Information

Below is the link to the electronic supplementary material.

Supplementary Material 1

## Data Availability

The datasets supporting the conclusions of this article are owned by the German statutory health insurance funds Barmer and Techniker Krankenkasse. Since public deposition of the data would breach ethical and legal compliance, data are only available upon formal request from the insurance funds. To fulfill the legal requirements to obtain that kind of data, researchers must obtain permission for a specific research question from the German Federal Office of Social Security. Additionally, researchers must conclude a contract with the statutory health insurance fund regarding data access. The licensee is permitted to use the data for the purpose of the research proposal within their company, exclusively. Thereby, a company is defined as an economic unit. Licensees are not allowed to pass the data to a third party, or to create software or databases except for scientific publications. Moreover, the study has to be approved by the data protection officer both at the statutory health insurance and the research institute.

## Abbreviations

ATC: Anatomical–Therapeutic–Chemical
CI: confidence interval
DSM: Diagnostic and Statistical Manual of Mental Disorders
GD: gender dysphoria
GI: gender incongruence
ICD: International Statistical Classification of Diseases and Related Health Problems
OPS: German procedure classification (“Operationen–und Prozedurenschlüssel”)
TK: Techniker Krankenkasse
