# Supplementary material for: Healthcare costs associated with gender dysphoria in children, adolescents and young adults in germany: A prevalence-based analysis using statutory health insurance data
Source: Eur J Health Econ. 2025 Aug 20;27(2):423–33. doi: 10.1007/s10198-025-01832-0 (PMC13046608; doi:10.1007/s10198-025-01832-0)
Supplement: Supplementary file 1 — Supplementary Material 1 [file 10198_2025_1832_MOESM1_ESM.docx]

# Supplemental material

### Table S 1 Additional indicators for diagnostic certainty to be included when coding ICD-10 diagnoses in the outpatient sector

| Indicator | Description |
| --- | --- |
| V | Suspected diagnosis (“Verdachtsdiagnose”) |
| G | Secured diagnosis (“gesicherte Diagnose”) |
| A | Excluded diagnosis (“ausgeschlossene Diagnose”) |
| Z | (Asymptomatic) condition following the respective diagnosis (“symptomloser Zustand nach der betreffenden Diagnose”) |

### Table S 2 Mean (variance; skewness) of selected covariates in individuals with prevalent GD vs. controls, before and after balancing on age, birth-assigned sex, and degree of urbanization.

|  | **2018** | | | **2019** | | | **2020** | | |
| --- | --- | --- | --- | --- | --- | --- | --- | --- | --- |
|  | GD  (n=2,837) | Control (before)  (n=97,073) | Control (after)  (n=97,073) | GD  (n=3,479) | Control (before)  (n=97,268) | Control (after)  (n=97,268) | GD  (n=4,086) | Control (before)  (n=95,962) | Control (after)  (n=95,962) |
| Age | 21.86 (31.03; -0.58) | 20.96 (39.09; -0.42) | 21.86 (31.03; -0.58) | 21.87 (30.12; -0.54) | 21.55 (36.54; -0.47) | 21.87 (30.12; -0.54) | 22.02 (28.16; -0.52) | 21.99 (33.61; -0.52) | 22.02 (28.16; -0.52) |
| Female birth-assigned sex | 0.67 (0.22; -0.74) | 0.72 (0.20; -1.00) | 0.67 (0.22; -0.74) | 0.66 (0.22; -0.69) | 0.72 (0.20; -0.98) | 0.66 (0.22; -0.69) | 0.65 (0.23; -0.65) | 0.72 (0.20; -0.95) | 0.65 (0.23; -0.65) |
| Urban county (ref: independent city) | 0.32 (0.22; 0.78) | 0.34 (0.22; 0.68) | 0.32 (0.22; 0.78) | 0.31 (0.21; 0.83) | 0.34 (0.22; 0.67) | 0.31 (0.21; 0.83) | 0.33 (0.22; 0.74) | 0.34 (0.23; 0.66) | 0.33 (0.22; 0.74) |
| Rural county | 0.13 (0.12; 2.16) | 0.13 (0.11; 2.19) | 0.13 (0.12; 2.16) | 0.13 (0.11; 2.22) | 0.13 (0.11; 2.20) | 0.13 (0.11; 2.22) | 0.11 (0.10; 2.43) | 0.13 (0.11; 2.18) | 0.11 (0.10; 2.42) |
| Sparsely populated county | 0.12 (0.10; 2.39) | 0.12 (0.11; 2.32) | 0.12 (0.10; 2.39) | 0.11 (0.10; 2.46) | 0.12 (0.11; 2.33) | 0.11 (0.10; 2.46) | 0.10 (0.09; 2.58) | 0.12 (0.11; 2.34) | 0.10 (0.09; 2.58) |
| ***Notes***: GD=gender dysphoria; ref.=reference group | | | | | | | | | |

### Table S 3 Mean (variance; skewness) of selected covariates in individuals with prevalent GD vs. controls, before and after balancing on age, birth-assigned sex, degree of urbanization, and psychiatric diagnoses.

|  | **2018** | | | **2019** | | | **2020** | | |
| --- | --- | --- | --- | --- | --- | --- | --- | --- | --- |
|  | GD  (n=2,837) | Control (before)  (n=97,073) | Control (after)  (n=97,073) | GD  (n=3,479) | Control (before)  (n=97,268) | Control (after)  (n=97,268) | GD  (n=4,086) | Control (before)  (n=95,962) | Control (after)  (n=95,962) |
| Age | 21.86 (31.03; -0.58) | 20.96 (39.09; -0.42) | 21.86 (31.03; -0.58) | 21.87 (30.12; -0.54) | 21.55 (36.54; -0.47) | 21.87 (30.12; -0.54) | 22.02 (28.16; -0.52) | 21.99 (33.61; -0.52) | 22.02 (28.16; -0.52) |
| Female birth-assigned sex | 0.67 (0.22; -0.74) | 0.72 (0.20; -1.00) | 0.67 (0.22; -0.74) | 0.66 (0.22; -0.69) | 0.72 (0.20; -0.98) | 0.66 (0.22; -0.69) | 0.65 (0.23; -0.65) | 0.72 (0.20; -0.95) | 0.65 (0.23; -0.65) |
| Urban county (ref: independent city) | 0.32 (0.22; 0.78) | 0.34 (0.22; 0.68) | 0.32 (0.22; 0.78) | 0.31 (0.21; 0.83) | 0.34 (0.22; 0.67) | 0.31 (0.21; 0.83) | 0.33 (0.22; 0.74) | 0.34 (0.23; 0.66) | 0.33 (0.22; 0.74) |
| Rural county | 0.13 (0.12; 2.16) | 0.13 (0.11; 2.19) | 0.13 (0.12; 2.16) | 0.13 (0.11; 2.22) | 0.13 (0.11; 2.20) | 0.13 (0.11; 2.22) | 0.11 (0.10; 2.43) | 0.13 (0.11; 2.18) | 0.11 (0.10; 2.42) |
| Sparsely populated county | 0.12 (0.10; 2.39) | 0.12 (0.11; 2.32) | 0.12 (0.10; 2.39) | 0.11 (0.10; 2.46) | 0.12 (0.11; 2.33) | 0.11 (0.10; 2.46) | 0.10 (0.09; 2.58) | 0.12 (0.11; 2.34) | 0.10 (0.09; 2.58) |
| Mental and behavioral disorders due to psychoactive substance use (F10-F19) | 0.08 (0.08; 3.00) | 0.03 (0.03; 5.32) | 0.08 (0.08; 3.00) | 0.09 (0.08; 2.97) | 0.03 (0.03; 5.08) | 0.09 (0.08; 2.97) | 0.08 (0.08; 3.00) | 0.03 (0.03; 5.35) | 0.08 (0.08; 3.00) |
| Affective disorders (F30-F39) | 0.42 (0.24; 0.31) | 0.08 (0.07; 3.10) | 0.42 (0.24; 0.31) | 0.44 (0.25; 0.23) | 0.09 (0.08; 2.96) | 0.44 (0.25; 0.23) | 0.47 (0.25; 0.10) | 0.09 (0.08; 2.88) | 0.47 (0.25; 0.10) |
| Reaction to severe stress, and adjustment disorders (F43) | 0.28 (0.20; 1.01) | 0.07 (0.07; 3.26) | 0.28 (0.20; 1.01) | 0.27 (0.20; 1.01) | 0.08 (0.07; 3.11) | 0.27 (0.20; 1.01) | 0.28 (0.20; 0.98) | 0.08 (0.07; 3.12) | 0.28 (0.20; 0.98) |
| Somatoform disorders (F45) | 0.14 (0.12; 2.03) | 0.07 (0.06; 3.41) | 0.14 (0.12; 2.03) | 0.14 (0.12; 2.11) | 0.07 (0.06; 3.42) | 0.14 (0.12; 2.11) | 0.14 (0.12; 2.09) | 0.07 (0.06; 3.47) | 0.14 (0.12; 2.09) |
| Phobic anxiety disorders (F40) | 0.09 (0.08; 2.86) | 0.02 (0.02; 7.65) | 0.09 (0.08; 2.86) | 0.10 (0.09; 2.69) | 0.02 (0.02; 7.39) | 0.10 (0.09; 2.69) | 0.11 (0.10; 2.52) | 0.02 (0.02; 7.18) | 0.11 (0.10; 2.51) |
| Other anxiety disorders (F41) | 0.14 (0.12; 2.06) | 0.04 (0.04; 4.69) | 0.14 (0.12; 2.06) | 0.14 (0.12; 2.07) | 0.04 (0.04; 4.47) | 0.14 (0.12; 2.07) | 0.15 (0.13; 1.92) | 0.05 (0.05; 4.25) | 0.15 (0.13; 1.92) |
| Other neurotic disorders (F48) | 0.05 (0.04; 4.38) | 0.02 (0.02; 6.62) | 0.05 (0.04; 4.38) | 0.04 (0.04; 4.68) | 0.02 (0.02; 6.48) | 0.04 (0.04; 4.68) | 0.03 (0.03; 5.08) | 0.02 (0.02; 6.80) | 0.03 (0.03; 5.08) |
| Eating disorders (F50) | 0.03 (0.03; 5.13) | 0.01 (0.01; 9.19) | 0.03 (0.03; 5.13) | 0.03 (0.03; 5.08) | 0.01 (0.01; 8.98) | 0.03 (0.03; 5.08) | 0.04 (0.03; 5.00) | 0.01 (0.01; 8.69) | 0.04 (0.03; 5.00) |
| Emotionally unstable personality disorder, type borderline (F60.31) | 0.07 (0.06; 3.45) | 0.01 (0.01; 11.18) | 0.07 (0.06; 3.45) | 0.07 (0.07; 3.36) | 0.01 (0.01; 10.51) | 0.07 (0.07; 3.36) | 0.08 (0.07; 3.17) | 0.01 (0.01; 10.27) | 0.08 (0.07; 3.17) |
| Hyperkinetic disorders (F90) | 0.07 (0.06; 3.40) | 0.02 (0.02; 6.31) | 0.07 (0.06; 3.40) | 0.08 (0.07; 3.18) | 0.02 (0.02; 6.35) | 0.08 (0.07; 3.18) | 0.08 (0.08; 3.03) | 0.02 (0.02; 6.30) | 0.08 (0.08; 3.03) |
| Other childhood emotional disorders (F93.8) | 0.04 (0.04; 4.59) | 0.01 (0.01; 11.27) | 0.04 (0.04; 4.59) | 0.03 (0.03; 5.20) | 0.01 (0.01; 11.10) | 0.03 (0.03; 5.20) | 0.04 (0.04; 4.58) | 0.01 (0.01; 11.38) | 0.04 (0.04; 4.58) |
| Other behavioral and emotional disorders with onset usually occurring in childhood and adolescence (F98) | 0.05 (0.04; 4.38) | 0.02 (0.02; 7.86) | 0.05 (0.04; 4.38) | 0.04 (0.04; 4.68) | 0.01 (0.01; 8.59) | 0.04 (0.04; 4.68) | 0.04 (0.04; 4.93) | 0.01 (0.01; 8.97) | 0.04 (0.04; 4.93) |
| Emotional disorders with onset specific to childhood (F93) | 0.02 (0.02; 6.54) | 0.01 (0.01; 10.94) | 0.02 (0.02; 6.54) | 0.02 (0.02; 6.12) | 0.01 (0.01; 11.55) | 0.02 (0.02; 6.12) | 0.02 (0.02; 6.19) | 0.01 (0.01; 12.16) | 0.02 (0.02; 6.19) |
| Acute and transient psychotic disorders (F23) | 0.00 (0.00; 26.58) | 0.00 (0.00; 34.15) | 0.00 (0.00; 26.57) | 0.00 (0.00; 20.78) | 0.00 (0.00; 34.83) | 0.00 (0.00; 20.78) | 0.00 (0.00; 15.89) | 0.00 (0.00; 33.56) | 0.00 (0.00; 15.89) |
| Dissociative [conversion] disorders (F44) | 0.02 (0.02; 7.66) | 0.00 (0.00; 21.03) | 0.02 (0.02; 7.66) | 0.02 (0.02; 7.23) | 0.00 (0.00; 20.67) | 0.02 (0.02; 7.23) | 0.02 (0.02; 7.39) | 0.00 (0.00; 20.35) | 0.02 (0.02; 7.39) |
| Conduct disorders (F91) | 0.02 (0.02; 6.13) | 0.01 (0.01; 12.35) | 0.02 (0.02; 6.13) | 0.02 (0.02; 6.28) | 0.01 (0.01; 12.31) | 0.02 (0.02; 6.28) | 0.02 (0.02; 6.59) | 0.01 (0.01; 13.10) | 0.02 (0.02; 6.59) |
| Mixed disorders of conduct and emotions (F92) | 0.02 (0.02; 6.27) | 0.00 (0.00; 15.68) | 0.02 (0.02; 6.27) | 0.02 (0.02; 6.20) | 0.00 (0.00; 16.17) | 0.02 (0.02; 6.20) | 0.02 (0.02; 6.59) | 0.00 (0.00; 16.54) | 0.02 (0.02; 6.59) |
| ***Notes***: GD=gender dysphoria; ref.=reference group | | | | | | | | | |

### Table S 4 Mean (variance; skewness) of selected covariates in individuals with prevalent GD and receiving hormonal therapy vs. controls, before and after balancing on age, birth-assigned sex, and degree of urbanization.

|  | **2018** | | | **2019** | | | **2020** | | |
| --- | --- | --- | --- | --- | --- | --- | --- | --- | --- |
|  | GD (n=1,570) | Control (before) (n=97,073) | Control (after) (n=97,073) | GD (n=2,031) | Control (before) (n=97,268) | Control (after) (n=97,268) | GD (n=2,567) | Control (before) (n=95,962) | Control (after) (n=95,962) |
| Age | 23.92 (17.14; -0.45) | 20.93 (39.09; -0.42) | 23.92 (17.13; -0.44) | 23.79 (17.23; -0.41) | 21.51 (36.61; -0.47) | 23.79 (17.23; -0.41) | 23.79 (16.55; -0.40) | 21.95 (33.74; -0.51) | 23.79 (16.55; -0.40) |
| Female birth-assigned sex | 0.69 (0.21; -0.83) | 0.72 (0.20; -0.99) | 0.69 (0.21; -0.83) | 0.69 (0.21; -0.81) | 0.72 (0.20; -0.97) | 0.69 (0.21; -0.81) | 0.69 (0.21; -0.83) | 0.71 (0.20; -0.94) | 0.69 (0.21; -0.83) |
| Urban county (ref: independent city) | 0.34 (0.22; 0.69) | 0.34 (0.22; 0.68) | 0.34 (0.22; 0.69) | 0.33 (0.22; 0.73) | 0.34 (0.22; 0.68) | 0.33 (0.22; 0.73) | 0.32 (0.22; 0.79) | 0.34 (0.23; 0.66) | 0.32 (0.22; 0.79) |
| Rural county | 0.10 (0.09; 2.73) | 0.13 (0.11; 2.18) | 0.10 (0.09; 2.73) | 0.09 (0.08; 2.84) | 0.13 (0.11; 2.19) | 0.09 (0.08; 2.84) | 0.10 (0.09; 2.58) | 0.13 (0.11; 2.18) | 0.10 (0.09; 2.58) |
| Sparsely populated county | 0.07 (0.07; 3.37) | 0.12 (0.11; 2.31) | 0.07 (0.07; 3.37) | 0.08 (0.07; 3.08) | 0.12 (0.11; 2.33) | 0.08 (0.07; 3.08) | 0.08 (0.08; 3.01) | 0.12 (0.11; 2.34) | 0.08 (0.08; 3.01) |
| ***Notes***: GD=gender dysphoria; ref.=reference group | | | | | | | | | |

### Table S 5 Mean (variance; skewness) of selected covariates in individuals with prevalent GD and receiving hormonal therapy vs. controls, before and after balancing on age, birth-assigned sex, degree of urbanization, and psychiatric diagnoses.

|  | **2018** | | | **2019** | | | **2020** | | |
| --- | --- | --- | --- | --- | --- | --- | --- | --- | --- |
|  | GD (n=1,570) | Control (before) (n=97,073) | Control (after) (n=97,073) | GD (n=2,031) | Control (before) (n=97,268) | Control (after) (n=97,268) | GD (n=2,567) | Control (before) (n=95,962) | Control (after) (n=95,962) |
| Age | 23.92 (17.14; -0.45) | 20.93 (39.09; -0.42) | 23.92 (17.13; -0.44) | 23.79 (17.23; -0.41) | 21.51 (36.61; -0.47) | 23.79 (17.23; -0.41) | 23.79 (16.55; -0.40) | 21.95 (33.74; -0.51) | 23.79 (16.55; -0.40) |
| Female birth-assigned sex | 0.69 (0.21; -0.83) | 0.72 (0.20; -0.99) | 0.69 (0.21; -0.83) | 0.69 (0.21; -0.81) | 0.72 (0.20; -0.97) | 0.69 (0.21; -0.81) | 0.69 (0.21; -0.83) | 0.71 (0.20; -0.94) | 0.69 (0.21; -0.83) |
| Urban county (ref: independent city) | 0.34 (0.22; 0.69) | 0.34 (0.22; 0.68) | 0.34 (0.22; 0.69) | 0.33 (0.22; 0.73) | 0.34 (0.22; 0.68) | 0.33 (0.22; 0.73) | 0.32 (0.22; 0.79) | 0.34 (0.23; 0.66) | 0.32 (0.22; 0.79) |
| Rural county | 0.10 (0.09; 2.73) | 0.13 (0.11; 2.18) | 0.10 (0.09; 2.73) | 0.09 (0.08; 2.84) | 0.13 (0.11; 2.19) | 0.09 (0.08; 2.84) | 0.10 (0.09; 2.58) | 0.13 (0.11; 2.18) | 0.10 (0.09; 2.58) |
| Sparsely populated county | 0.07 (0.07; 3.37) | 0.12 (0.11; 2.31) | 0.07 (0.07; 3.37) | 0.08 (0.07; 3.08) | 0.12 (0.11; 2.33) | 0.08 (0.07; 3.08) | 0.08 (0.08; 3.01) | 0.12 (0.11; 2.34) | 0.08 (0.08; 3.01) |
| Mental and behavioral disorders due to psychoactive substance use (F10-F19) | 0.09 (0.08; 2.82) | 0.03 (0.03; 5.27) | 0.09 (0.08; 2.82) | 0.09 (0.08; 2.96) | 0.04 (0.03; 5.02) | 0.09 (0.08; 2.96) | 0.09 (0.08; 2.87) | 0.03 (0.03; 5.28) | 0.09 (0.08; 2.87) |
| Affective disorders (F30-F39) | 0.43 (0.25; 0.26) | 0.08 (0.08; 3.00) | 0.43 (0.25; 0.26) | 0.44 (0.25; 0.23) | 0.09 (0.08; 2.84) | 0.44 (0.25; 0.23) | 0.46 (0.25; 0.17) | 0.10 (0.09; 2.75) | 0.46 (0.25; 0.17) |
| Reaction to severe stress, and adjustment disorders (F43) | 0.26 (0.19; 1.07) | 0.08 (0.07; 3.18) | 0.26 (0.19; 1.07) | 0.26 (0.19; 1.08) | 0.08 (0.08; 3.03) | 0.26 (0.19; 1.08) | 0.25 (0.19; 1.14) | 0.08 (0.08; 3.03) | 0.25 (0.19; 1.14) |
| Somatoform disorders (F45) | 0.12 (0.11; 2.28) | 0.07 (0.06; 3.38) | 0.12 (0.11; 2.28) | 0.14 (0.12; 2.13) | 0.07 (0.06; 3.38) | 0.14 (0.12; 2.13) | 0.13 (0.11; 2.24) | 0.07 (0.06; 3.43) | 0.13 (0.11; 2.23) |
| Phobic anxiety disorders (F40) | 0.08 (0.07; 3.14) | 0.02 (0.02; 7.38) | 0.08 (0.07; 3.14) | 0.09 (0.08; 2.88) | 0.02 (0.02; 7.09) | 0.09 (0.08; 2.88) | 0.10 (0.09; 2.72) | 0.02 (0.02; 6.85) | 0.10 (0.09; 2.72) |
| Other anxiety disorders (F41) | 0.14 (0.12; 2.13) | 0.04 (0.04; 4.60) | 0.14 (0.12; 2.13) | 0.14 (0.12; 2.06) | 0.04 (0.04; 4.39) | 0.14 (0.12; 2.06) | 0.14 (0.12; 2.06) | 0.05 (0.05; 4.15) | 0.14 (0.12; 2.06) |
| Other neurotic disorders (F48) | 0.04 (0.04; 4.53) | 0.02 (0.02; 6.56) | 0.04 (0.04; 4.52) | 0.04 (0.04; 4.84) | 0.02 (0.02; 6.44) | 0.04 (0.04; 4.84) | 0.04 (0.04; 4.91) | 0.02 (0.02; 6.77) | 0.04 (0.04; 4.90) |
| Eating disorders (F50) | 0.03 (0.03; 5.45) | 0.01 (0.01; 9.05) | 0.03 (0.03; 5.45) | 0.03 (0.03; 5.36) | 0.01 (0.01; 8.82) | 0.03 (0.03; 5.36) | 0.03 (0.03; 5.59) | 0.01 (0.01; 8.51) | 0.03 (0.03; 5.59) |
| Emotionally unstable personality disorder, type borderline (F60.31) | 0.07 (0.06; 3.45) | 0.01 (0.01; 10.66) | 0.07 (0.06; 3.45) | 0.07 (0.07; 3.23) | 0.01 (0.01; 10.04) | 0.07 (0.07; 3.23) | 0.08 (0.07; 3.08) | 0.01 (0.01; 9.76) | 0.08 (0.07; 3.08) |
| Hyperkinetic disorders (F90) | 0.04 (0.04; 4.41) | 0.02 (0.02; 6.17) | 0.04 (0.04; 4.41) | 0.05 (0.05; 4.01) | 0.02 (0.02; 6.17) | 0.05 (0.05; 4.00) | 0.06 (0.06; 3.74) | 0.03 (0.02; 6.08) | 0.06 (0.06; 3.74) |
| Other childhood emotional disorders (F93.8) | 0.02 (0.02; 7.58) | 0.01 (0.01; 10.69) | 0.02 (0.02; 7.58) | 0.01 (0.01; 8.50) | 0.01 (0.01; 10.57) | 0.01 (0.01; 8.50) | 0.01 (0.01; 8.04) | 0.01 (0.01; 10.52) | 0.01 (0.01; 8.03) |
| Other behavioral and emotional disorders with onset usually occurring in childhood and adolescence (F98) | 0.02 (0.02; 7.15) | 0.02 (0.02; 7.65) | 0.02 (0.02; 7.15) | 0.02 (0.02; 7.31) | 0.01 (0.01; 8.30) | 0.02 (0.02; 7.31) | 0.01 (0.01; 8.27) | 0.01 (0.01; 8.61) | 0.01 (0.01; 8.27) |
| Emotional disorders with onset specific to childhood (F93) | 0.01 (0.01; 11.31) | 0.01 (0.01; 10.67) | 0.01 (0.01; 11.30) | 0.01 (0.01; 11.13) | 0.01 (0.01; 11.09) | 0.01 (0.01; 11.13) | 0.01 (0.01; 10.66) | 0.01 (0.01; 11.56) | 0.01 (0.01; 10.66) |
| Acute and transient psychotic disorders (F23) | 0.00 (0.00; 39.59) | 0.00 (0.00; 33.77) | 0.00 (0.00; 39.58) | 0.00 (0.00; 20.08) | 0.00 (0.00; 34.44) | 0.00 (0.00; 20.08) | 0.00 (0.00; 19.07) | 0.00 (0.00; 32.16) | 0.00 (0.00; 19.07) |
| Dissociative [conversion] disorders (F44) | 0.02 (0.02; 7.43) | 0.00 (0.00; 20.30) | 0.02 (0.02; 7.43) | 0.02 (0.02; 7.01) | 0.00 (0.00; 19.80) | 0.02 (0.02; 7.01) | 0.02 (0.02; 7.19) | 0.00 (0.00; 19.51) | 0.02 (0.02; 7.19) |
| Conduct disorders (F91) | 0.01 (0.01; 13.09) | 0.01 (0.01; 11.86) | 0.01 (0.01; 13.09) | 0.01 (0.01; 12.38) | 0.01 (0.01; 11.77) | 0.01 (0.01; 12.38) | 0.01 (0.01; 11.49) | 0.01 (0.01; 12.43) | 0.01 (0.01; 11.49) |
| Mixed disorders of conduct and emotions (F92) | 0.01 (0.01; 12.41) | 0.00 (0.00; 14.73) | 0.01 (0.01; 12.41) | 0.01 (0.01; 10.48) | 0.00 (0.00; 14.98) | 0.01 (0.01; 10.48) | 0.01 (0.01; 11.82) | 0.00 (0.00; 15.19) | 0.01 (0.01; 11.81) |
| ***Notes***: GD=gender dysphoria; ref.=reference group | | | | | | | | | |

### Table S 6 Mean costs and resource use 2019 for individuals with prevalent GD vs. controls, total and by sectors, age group, and birth-assigned sex.

|  | | **Total sample** | | | **By age group** | | | | | | | | | | | | **By birth-assigned sex** | | | | | |
| --- | --- | --- | --- | --- | --- | --- | --- | --- | --- | --- | --- | --- | --- | --- | --- | --- | --- | --- | --- | --- | --- | --- |
|  |  |  |  |  | 4-12 years | | | 13-17 years | | | 18-24 years | | | 25-30 years | | | male | | | female | | |
|  |  | GD (n=3,479) | Control (n=97,268) | | GD (n=168) | Control (n=7,866) | | GD (n=599) | Control (n=19,004) | | GD (n=1433) | Control (n=32,499) | | GD (n=1,279) | Control (n=37,899) | | GD (n=1,169) | Control (n=27,274) | | GD (n=2,310) | Control (n=69,994) | |
|  |  |  | (1) | (2) |  | (1) | (2) |  | (1) | (2) |  | (1) | (2) |  | (1) | (2) |  | (1) | (2) |  | (1) | (2) |
| **Cost** |  |  |  |  |  |  |  |  |  |  |  |  |  |  |  |  |  |  |  |  |  |  |
| **Total** | Mean | **5978** | **1134** | **3225** | **3123** | **837** | **2271** | **8076** | **1148** | **4921** | **6079** | **1060** | **3058** | **5256** | **1248** | **2745** | **6131** | **920** | **2679** | **5900** | **1243** | **3501** |
|  | SD | 16141 | 4968 | 9575 | 6900 | 5822 | 8318 | 15382 | 5443 | 14462 | 21539 | 4140 | 7647 | 8283 | 5365 | 8545 | 24197 | 5369 | 9498 | 9812 | 4748 | 9602 |
|  | Prop. | 1.00 | 0.89 | 0.94 | 1.00 | 0.91 | 0.93 | 1.00 | 0.89 | 0.93 | 1.00 | 0.89 | 0.94 | 1.00 | 0.90 | 0.95 | 1.00 | 0.84 | 0.90 | 1.00 | 0.92 | 0.96 |
| **Outpatient** | Mean | **1400** | **412** | **855** | **1103** | **335** | **553** | **1773** | **375** | **922** | **1434** | **385** | **820** | **1227** | **469** | **903** | **1405** | **288** | **627** | **1398** | **475** | **971** |
|  | SD | 1366 | 755 | 1251 | 1226 | 599 | 882 | 1585 | 698 | 1333 | 1385 | 759 | 1242 | 1204 | 792 | 1259 | 1399 | 733 | 1143 | 1350 | 758 | 1287 |
|  | Prop. | 1.00 | 0.89 | 0.94 | 1.00 | 0.90 | 0.93 | 1.00 | 0.88 | 0.93 | 1.00 | 0.88 | 0.94 | 1.00 | 0.90 | 0.94 | 1.00 | 0.83 | 0.90 | 1.00 | 0.92 | 0.96 |
| **GP** | Mean | **210** | **105** | **155** | **220** | **115** | **139** | **231** | **101** | **143** | **203** | **107** | **161** | **208** | **105** | **155** | **202** | **94** | **137** | **215** | **111** | **164** |
|  | SD | 197 | 124 | 169 | 149 | 126 | 141 | 236 | 117 | 142 | 184 | 117 | 164 | 197 | 132 | 187 | 173 | 114 | 147 | 208 | 128 | 178 |
|  | Prop. | 0.94 | 0.77 | 0.85 | 0.95 | 0.79 | 0.83 | 0.95 | 0.75 | 0.83 | 0.94 | 0.78 | 0.87 | 0.93 | 0.77 | 0.85 | 0.93 | 0.73 | 0.81 | 0.95 | 0.79 | 0.87 |
| **Psych.** | Mean | **811** | **112** | **456** | **688** | **120** | **296** | **1259** | **146** | **619** | **848** | **90** | **413** | **575** | **117** | **448** | **848** | **84** | **347** | **791** | **126** | **511** |
|  | SD | 1258 | 548 | 1071 | 1142 | 520 | 798 | 1503 | 607 | 1230 | 1281 | 478 | 1000 | 1042 | 588 | 1089 | 1297 | 480 | 952 | 1237 | 579 | 1123 |
|  | Prop. | 0.61 | 0.10 | 0.33 | 0.49 | 0.10 | 0.23 | 0.70 | 0.11 | 0.36 | 0.64 | 0.09 | 0.32 | 0.54 | 0.10 | 0.33 | 0.62 | 0.08 | 0.27 | 0.60 | 0.11 | 0.36 |
| **Other** | Mean | **379** | **195** | **245** | **195** | **100** | **118** | **283** | **128** | **160** | **384** | **188** | **247** | **444** | **248** | **300** | **354** | **110** | **143** | **392** | **238** | **296** |
|  | SD | 457 | 442 | 476 | 263 | 193 | 222 | 357 | 214 | 255 | 468 | 525 | 567 | 492 | 445 | 469 | 427 | 504 | 516 | 472 | 400 | 446 |
|  | Prop. | 0.92 | 0.76 | 0.82 | 0.79 | 0.67 | 0.71 | 0.88 | 0.72 | 0.78 | 0.93 | 0.76 | 0.82 | 0.94 | 0.79 | 0.85 | 0.88 | 0.61 | 0.68 | 0.94 | 0.84 | 0.89 |
| **Hospital inpatient** | Mean | **3578** | **456** | **1864** | **1417** | **312** | **1330** | **5184** | **489** | **3459** | **3628** | **444** | **1816** | **3052** | **474** | **1250** | **3633** | **386** | **1539** | **3550** | **491** | **2028** |
|  | SD | 15433 | 3506 | 8255 | 6235 | 5580 | 7696 | 14746 | 4049 | 13536 | 20735 | 2810 | 6613 | 7583 | 3488 | 6301 | 23436 | 4251 | 7880 | 8996 | 3059 | 8434 |
|  | Prop. | 0.31 | 0.10 | 0.19 | 0.10 | 0.06 | 0.10 | 0.32 | 0.08 | 0.20 | 0.35 | 0.10 | 0.21 | 0.30 | 0.11 | 0.18 | 0.25 | 0.08 | 0.15 | 0.34 | 0.11 | 0.21 |
| Psych. | Mean | **1687** | **171** | **1397** | **1134** | **88** | **1057** | **4336** | **264** | **3049** | **1405** | **183** | **1342** | **835** | **127** | **734** | **1852** | **141** | **1117** | **1604** | **185** | **1538** |
|  | SD | 8069 | 2314 | 7278 | 5637 | 1708 | 5908 | 14167 | 3631 | 13246 | 6827 | 2190 | 5785 | 4768 | 1639 | 4145 | 9211 | 2040 | 5937 | 7425 | 2441 | 7867 |
|  | Prop. | 0.09 | 0.01 | 0.08 | 0.05 | 0.00 | 0.04 | 0.17 | 0.01 | 0.11 | 0.10 | 0.01 | 0.10 | 0.06 | 0.01 | 0.06 | 0.10 | 0.01 | 0.07 | 0.09 | 0.01 | 0.09 |
| Non-psych. | Mean | **1890** | **285** | **467** | **283** | **224** | **273** | **848** | **225** | **410** | **2223** | **261** | **473** | **2217** | **347** | **516** | **1780** | **245** | **422** | **1946** | **306** | **490** |
|  | SD | 13265 | 2552 | 3352 | 2020 | 5315 | 4985 | 3729 | 1708 | 2287 | 19692 | 1669 | 2232 | 6021 | 2970 | 4307 | 21658 | 3629 | 4742 | 5270 | 1773 | 2354 |
|  | Prop. | 0.24 | 0.09 | 0.14 | 0.06 | 0.05 | 0.07 | 0.19 | 0.07 | 0.13 | 0.28 | 0.09 | 0.15 | 0.26 | 0.11 | 0.14 | 0.18 | 0.07 | 0.10 | 0.28 | 0.10 | 0.16 |
| **Hospital outpatient** | Mean | **312** | **47** | **159** | **324** | **65** | **208** | **539** | **65** | **251** | **279** | **40** | **142** | **240** | **44** | **128** | **324** | **42** | **127** | **305** | **50** | **176** |
|  | SD | 765 | 287 | 578 | 525 | 301 | 755 | 1082 | 321 | 668 | 661 | 261 | 540 | 700 | 294 | 535 | 771 | 240 | 461 | 763 | 308 | 628 |
|  | Prop. | 0.35 | 0.08 | 0.19 | 0.45 | 0.11 | 0.19 | 0.52 | 0.10 | 0.27 | 0.33 | 0.07 | 0.18 | 0.28 | 0.08 | 0.16 | 0.38 | 0.07 | 0.17 | 0.34 | 0.08 | 0.20 |
| Psych. | Mean | **160** | **12** | **69** | **183** | **13** | **48** | **334** | **22** | **143** | **118** | **11** | **62** | **121** | **10** | **45** | **169** | **12** | **57** | **155** | **13** | **75** |
|  | SD | 557 | 121 | 296 | 375 | 120 | 226 | 854 | 168 | 451 | 377 | 104 | 260 | 554 | 111 | 237 | 562 | 116 | 280 | 555 | 123 | 304 |
|  | Prop. | 0.19 | 0.02 | 0.10 | 0.27 | 0.02 | 0.07 | 0.37 | 0.03 | 0.17 | 0.16 | 0.02 | 0.10 | 0.11 | 0.02 | 0.07 | 0.20 | 0.02 | 0.09 | 0.18 | 0.02 | 0.11 |
| Somatic | Mean | **152** | **35** | **91** | **141** | **52** | **160** | **206** | **43** | **108** | **161** | **30** | **81** | **119** | **35** | **83** | **155** | **30** | **70** | **151** | **38** | **101** |
|  | SD | 516 | 255 | 482 | 408 | 272 | 716 | 657 | 264 | 458 | 529 | 233 | 456 | 431 | 269 | 476 | 546 | 207 | 349 | 500 | 276 | 536 |
|  | Prop. | 0.22 | 0.06 | 0.11 | 0.24 | 0.09 | 0.15 | 0.28 | 0.08 | 0.14 | 0.22 | 0.05 | 0.10 | 0.20 | 0.06 | 0.11 | 0.24 | 0.06 | 0.10 | 0.22 | 0.07 | 0.12 |
| **Medication** | Mean | **688** | **219** | **346** | **279** | **125** | **180** | **580** | **219** | **289** | **738** | **191** | **280** | **737** | **261** | **465** | **770** | **205** | **386** | **647** | **226** | **326** |
|  | SD | 3755 | 3022 | 3587 | 1470 | 895 | 1076 | 3053 | 3082 | 2607 | 5069 | 2538 | 2358 | 2216 | 3597 | 4990 | 5361 | 2770 | 4215 | 2588 | 3142 | 3223 |
|  | Prop. | 0.91 | 0.63 | 0.73 | 0.89 | 0.69 | 0.74 | 0.84 | 0.59 | 0.69 | 0.92 | 0.65 | 0.75 | 0.94 | 0.61 | 0.72 | 0.89 | 0.52 | 0.63 | 0.92 | 0.68 | 0.78 |
| Psych. | Mean | **51** | **11** | **47** | **30** | **15** | **47** | **60** | **13** | **49** | **42** | **9** | **46** | **60** | **11** | **47** | **68** | **14** | **54** | **43** | **9** | **44** |
|  | SD | 216 | 108 | 237 | 130 | 127 | 241 | 226 | 117 | 251 | 162 | 96 | 231 | 267 | 109 | 235 | 289 | 145 | 303 | 167 | 89 | 194 |
|  | Prop. | 0.25 | 0.05 | 0.22 | 0.09 | 0.03 | 0.10 | 0.23 | 0.04 | 0.17 | 0.26 | 0.06 | 0.24 | 0.26 | 0.06 | 0.23 | 0.27 | 0.05 | 0.19 | 0.23 | 0.06 | 0.23 |
| Somatic | Mean | **637** | **208** | **299** | **249** | **110** | **133** | **520** | **205** | **240** | **695** | **182** | **233** | **677** | **251** | **418** | **702** | **190** | **332** | **604** | **217** | **282** |
|  | SD | 3746 | 3018 | 3576 | 1465 | 879 | 1047 | 3045 | 3078 | 2593 | 5067 | 2534 | 2343 | 2184 | 3593 | 4981 | 5351 | 2763 | 4199 | 2579 | 3140 | 3214 |
|  | Prop. | 0.89 | 0.62 | 0.70 | 0.88 | 0.69 | 0.74 | 0.81 | 0.59 | 0.67 | 0.89 | 0.64 | 0.72 | 0.92 | 0.60 | 0.68 | 0.86 | 0.51 | 0.60 | 0.90 | 0.67 | 0.75 |
| **Resource use** |  |  |  |  |  |  |  |  |  |  |  |  |  |  |  |  |  |  |  |  |  |  |
| **Days in hospital** | Mean | **7.21** | **0.96** | **5.02** | **2.68** | **0.41** | **2.65** | **11.17** | **0.94** | **7.39** | **7.25** | **1.06** | **5.65** | **5.92** | **0.96** | **3.61** | **7.51** | **0.78** | **4.07** | **7.06** | **1.06** | **5.50** |
|  | SD | 23.27 | 7.86 | 20.81 | 13.25 | 4.41 | 13.40 | 31.93 | 8.80 | 27.47 | 23.02 | 8.50 | 21.64 | 19.19 | 7.07 | 16.68 | 27.06 | 7.65 | 18.26 | 21.09 | 7.96 | 21.98 |
|  | Prop. | 0.31 | 0.10 | 0.19 | 0.10 | 0.06 | 0.10 | 0.32 | 0.08 | 0.20 | 0.35 | 0.10 | 0.21 | 0.30 | 0.11 | 0.18 | 0.26 | 0.08 | 0.15 | 0.34 | 0.11 | 0.21 |
| Psych. | Mean | **4.93** | **0.57** | **4.28** | **2.40** | **0.21** | **2.32** | **9.73** | **0.61** | **6.55** | **4.79** | **0.67** | **4.88** | **3.17** | **0.50** | **2.89** | **5.66** | **0.48** | **3.50** | **4.56** | **0.61** | **4.67** |
|  | SD | 21.48 | 7.18 | 19.81 | 12.46 | 3.97 | 13.02 | 30.49 | 8.13 | 26.59 | 20.50 | 7.90 | 20.60 | 17.74 | 6.24 | 15.49 | 24.79 | 6.85 | 17.25 | 19.59 | 7.34 | 20.97 |
|  | Prop. | 0.09 | 0.01 | 0.08 | 0.05 | 0.00 | 0.04 | 0.17 | 0.01 | 0.11 | 0.10 | 0.01 | 0.10 | 0.06 | 0.01 | 0.06 | 0.10 | 0.01 | 0.07 | 0.09 | 0.01 | 0.09 |
| Somatic | Mean | **2.28** | **0.39** | **0.74** | **0.28** | **0.20** | **0.33** | **1.44** | **0.33** | **0.85** | **2.45** | **0.39** | **0.77** | **2.75** | **0.46** | **0.71** | **1.85** | **0.29** | **0.57** | **2.50** | **0.44** | **0.82** |
|  | SD | 9.12 | 2.91 | 4.82 | 1.78 | 1.93 | 3.39 | 8.60 | 3.17 | 6.43 | 10.87 | 2.87 | 4.10 | 7.61 | 2.93 | 4.80 | 11.24 | 3.09 | 4.97 | 7.83 | 2.81 | 4.73 |
|  | Prop. | 0.25 | 0.09 | 0.14 | 0.07 | 0.05 | 0.07 | 0.19 | 0.07 | 0.13 | 0.28 | 0.09 | 0.15 | 0.26 | 0.11 | 0.14 | 0.18 | 0.07 | 0.10 | 0.28 | 0.10 | 0.16 |
| **DDD** | Mean | **466** | **122** | **209** | **156** | **127** | **171** | **296** | **142** | **199** | **447** | **132** | **220** | **606** | **101** | **207** | **593** | **88** | **159** | **401** | **139** | **234** |
|  | SD | 487 | 268 | 392 | 208 | 289 | 437 | 386 | 294 | 335 | 409 | 252 | 365 | 580 | 268 | 433 | 621 | 252 | 374 | 387 | 275 | 398 |
|  | Prop. | 0.91 | 0.62 | 0.72 | 0.88 | 0.68 | 0.73 | 0.83 | 0.59 | 0.69 | 0.91 | 0.64 | 0.75 | 0.93 | 0.60 | 0.72 | 0.88 | 0.51 | 0.63 | 0.92 | 0.67 | 0.77 |
| Psych. | Mean | **74** | **13** | **65** | **12** | **8** | **23** | **56** | **9** | **42** | **77** | **12** | **68** | **87** | **16** | **78** | **85** | **12** | **52** | **69** | **13** | **71** |
|  | SD | 196 | 85 | 201 | 52 | 57 | 94 | 155 | 64 | 131 | 194 | 79 | 195 | 223 | 100 | 241 | 212 | 82 | 199 | 187 | 86 | 202 |
|  | Prop. | 0.25 | 0.05 | 0.22 | 0.09 | 0.03 | 0.10 | 0.23 | 0.04 | 0.17 | 0.26 | 0.06 | 0.24 | 0.26 | 0.06 | 0.23 | 0.27 | 0.05 | 0.19 | 0.23 | 0.06 | 0.23 |
| Somatic | Mean | **391** | **109** | **144** | **143** | **119** | **148** | **240** | **133** | **157** | **370** | **120** | **152** | **519** | **85** | **129** | **508** | **76** | **107** | **332** | **126** | **162** |
|  | SD | 431 | 247 | 303 | 200 | 281 | 416 | 347 | 284 | 299 | 360 | 232 | 272 | 513 | 236 | 316 | 569 | 228 | 282 | 325 | 254 | 312 |
|  | Prop. | 0.88 | 0.61 | 0.68 | 0.86 | 0.68 | 0.72 | 0.78 | 0.58 | 0.65 | 0.89 | 0.63 | 0.71 | 0.91 | 0.59 | 0.67 | 0.84 | 0.50 | 0.58 | 0.89 | 0.66 | 0.74 |
| ***Notes***: Control (1) = balanced for age, birth-assigned sex, and degree of urbanization; Control (2) = balanced for age, birth-assigned sex, degree of urbanization, and psychiatric diagnoses; DDD=defined daily dosed; GD=gender dysphoria; GP=general practitioner; Prop.=proportion of users; Psych.=psychiatric; SD=standard deviation. | | | | | | | | | | | | | | | | | | | | | | |

### Table S 7 Mean costs and resource use 2019 for individuals with prevalent GD and receiving hormonal therapy vs. controls, total and by sectors, age group, and birth-assigned sex.

|  | | **Total sample** | | | **By age group** | | | | | | | | | | | | **By birth-assigned sex** | | | | | |
| --- | --- | --- | --- | --- | --- | --- | --- | --- | --- | --- | --- | --- | --- | --- | --- | --- | --- | --- | --- | --- | --- | --- |
|  |  |  |  |  | 4-12 years | | | 13-17 years | | | 18-24 years | | | 25-30 years | | | male | | | female | | |
|  |  | GD (n=2,031) | Control (n=97,268) | | GD (n=9) | Control (n=7,866) | | GD (n=140) | Control (n=19,004) | | GD (n=916) | Control (n=32,499) | | GD (n=966) | Control (n=37,899) | | GD (n=633) | Control (n=27,274) | | GD (n=1,398) | Control (n=69,994) | |
|  |  |  | (1) | (2) |  | (1) | (2) |  | (1) | (2) |  | (1) | (2) |  | (1) | (2) |  | (1) | (2) |  | (1) | (2) |
| **Cost** |  |  |  |  |  |  |  |  |  |  |  |  |  |  |  |  |  |  |  |  |  |  |
| **Total** | Mean | **6347** | **1166** | **2747** | **8239** | **807** | **1941** | **9203** | **1205** | **4000** | **6634** | **1063** | **2709** | **5643** | **1256** | **2576** | **6690** | **918** | **2270** | **6192** | **1278** | **2963** |
|  | SD | 18164 | 4922 | 8107 | 10542 | 3615 | 6661 | 11837 | 5669 | 11808 | 25267 | 4094 | 6978 | 8114 | 5467 | 8310 | 30087 | 5293 | 8824 | 8357 | 4740 | 7750 |
|  | Prop. | 1.00 | 0.90 | 0.94 | 1.00 | 0.89 | 0.92 | 1.00 | 0.89 | 0.92 | 1.00 | 0.89 | 0.93 | 1.00 | 0.90 | 0.94 | 1.00 | 0.83 | 0.89 | 1.00 | 0.92 | 0.96 |
| **Outpatient** | Mean | **1407** | **429** | **819** | **1550** | **339** | **521** | **2196** | **393** | **880** | **1480** | **387** | **758** | **1222** | **475** | **868** | **1458** | **276** | **567** | **1384** | **499** | **934** |
|  | SD | 1329 | 769 | 1222 | 1411 | 631 | 860 | 1822 | 740 | 1340 | 1336 | 739 | 1176 | 1182 | 800 | 1242 | 1414 | 734 | 1120 | 1288 | 775 | 1248 |
|  | Prop. | 1.00 | 0.89 | 0.93 | 1.00 | 0.88 | 0.91 | 1.00 | 0.88 | 0.92 | 1.00 | 0.88 | 0.93 | 1.00 | 0.90 | 0.94 | 1.00 | 0.82 | 0.88 | 1.00 | 0.92 | 0.96 |
| GP | Mean | **216** | **105** | **149** | **324** | **103** | **120** | **258** | **101** | **135** | **217** | **107** | **153** | **209** | **105** | **149** | **198** | **90** | **127** | **225** | **112** | **160** |
|  | SD | 184 | 124 | 167 | 131 | 127 | 141 | 149 | 116 | 136 | 191 | 117 | 160 | 181 | 131 | 177 | 153 | 110 | 141 | 195 | 129 | 176 |
|  | Prop. | 0.94 | 0.77 | 0.85 | 1.00 | 0.75 | 0.79 | 0.99 | 0.75 | 0.82 | 0.95 | 0.78 | 0.86 | 0.94 | 0.77 | 0.84 | 0.93 | 0.72 | 0.80 | 0.95 | 0.80 | 0.87 |
| Psych. | Mean | **721** | **110** | **410** | **763** | **139** | **290** | **1486** | **157** | **580** | **804** | **91** | **365** | **532** | **121** | **425** | **781** | **74** | **296** | **694** | **127** | **462** |
|  | SD | 1161 | 554 | 1036 | 1174 | 558 | 782 | 1678 | 642 | 1229 | 1181 | 478 | 950 | 984 | 600 | 1073 | 1247 | 470 | 914 | 1119 | 587 | 1082 |
|  | Prop. | 0.60 | 0.10 | 0.30 | 0.56 | 0.12 | 0.23 | 0.80 | 0.11 | 0.32 | 0.65 | 0.09 | 0.30 | 0.51 | 0.10 | 0.31 | 0.60 | 0.07 | 0.24 | 0.59 | 0.11 | 0.33 |
| Other | Mean | **469** | **214** | **259** | **463** | **97** | **111** | **452** | **135** | **165** | **459** | **190** | **239** | **481** | **250** | **295** | **478** | **112** | **143** | **465** | **260** | **312** |
|  | SD | 498 | 455 | 486 | 299 | 172 | 188 | 371 | 227 | 266 | 518 | 497 | 536 | 496 | 441 | 463 | 473 | 514 | 535 | 509 | 417 | 452 |
|  | Prop. | 0.97 | 0.78 | 0.83 | 1.00 | 0.68 | 0.70 | 0.96 | 0.73 | 0.78 | 0.97 | 0.77 | 0.82 | 0.97 | 0.80 | 0.84 | 0.97 | 0.60 | 0.66 | 0.97 | 0.86 | 0.90 |
| **Hospital inpatient** | Mean | **3813** | **463** | **1447** | **2494** | **288** | **1087** | **5199** | **519** | **2633** | **4079** | **446** | **1556** | **3372** | **469** | **1147** | **4156** | **398** | **1223** | **3658** | **492** | **1548** |
|  | SD | 17686 | 3267 | 6547 | 7483 | 3202 | 6001 | 11359 | 4084 | 10761 | 24824 | 2841 | 5937 | 7351 | 3477 | 6118 | 29843 | 4064 | 7053 | 7177 | 2833 | 6302 |
|  | Prop. | 0.36 | 0.11 | 0.18 | 0.11 | 0.05 | 0.10 | 0.45 | 0.08 | 0.18 | 0.40 | 0.10 | 0.19 | 0.32 | 0.11 | 0.17 | 0.29 | 0.08 | 0.14 | 0.40 | 0.12 | 0.20 |
| Psych. | Mean | **900** | **164** | **983** | **2494** | **121** | **874** | **3520** | **284** | **2230** | **767** | **184** | **1118** | **631** | **125** | **648** | **1137** | **149** | **788** | **793** | **170** | **1072** |
|  | SD | 4961 | 2126 | 5287 | 7483 | 2144 | 5468 | 11254 | 3674 | 10437 | 4071 | 2216 | 5183 | 4031 | 1627 | 3871 | 5921 | 2090 | 4385 | 4457 | 2142 | 5646 |
|  | Prop. | 0.07 | 0.01 | 0.07 | 0.11 | 0.01 | 0.04 | 0.18 | 0.01 | 0.09 | 0.07 | 0.01 | 0.08 | 0.05 | 0.01 | 0.05 | 0.08 | 0.10 | 0.06 | 0.09 | 0.09 | 0.07 |
| Somatic | Mean | **2913** | **299** | **464** | **0** | **167** | **212** | **1679** | **235** | **403** | **3312** | **263** | **438** | **2741** | **344** | **499** | **3020** | **249** | **435** | **2865** | **322** | **476** |
|  | SD | 17077 | 2386 | 3358 | 0 | 2385 | 2540 | 3065 | 1688 | 2219 | 24554 | 1689 | 2165 | 6318 | 2968 | 4303 | 29341 | 3344 | 4954 | 5855 | 1790 | 2295 |
|  | Prop. | 0.32 | 0.10 | 0.14 | 0.00 | 0.05 | 0.06 | 0.31 | 0.07 | 0.12 | 0.35 | 0.09 | 0.14 | 0.30 | 0.11 | 0.13 | 0.24 | 0.07 | 0.10 | 0.36 | 0.11 | 0.15 |
| **Hospital outpatient** | Mean | **277** | **45** | **132** | **807** | **53** | **158** | **729** | **67** | **209** | **281** | **40** | **128** | **204** | **45** | **123** | **287** | **35** | **95** | **273** | **49** | **149** |
|  | SD | 714 | 289 | 534 | 615 | 276 | 670 | 1541 | 332 | 602 | 641 | 265 | 518 | 547 | 302 | 535 | 654 | 225 | 386 | 739 | 313 | 588 |
|  | Prop. | 0.34 | 0.08 | 0.16 | 0.89 | 0.10 | 0.17 | 0.69 | 0.10 | 0.23 | 0.34 | 0.07 | 0.16 | 0.28 | 0.08 | 0.15 | 0.37 | 0.06 | 0.14 | 0.32 | 0.08 | 0.18 |
| Psych. | Mean | **119** | **11** | **52** | **490** | **14** | **46** | **473** | **23** | **116** | **107** | **10** | **52** | **77** | **10** | **42** | **120** | **10** | **41** | **119** | **11** | **57** |
|  | SD | 513 | 114 | 248 | 642 | 120 | 215 | 1396 | 174 | 395 | 384 | 101 | 232 | 328 | 112 | 229 | 394 | 106 | 213 | 558 | 117 | 263 |
|  | Prop. | 0.14 | 0.02 | 0.08 | 0.44 | 0.02 | 0.06 | 0.44 | 0.03 | 0.14 | 0.14 | 0.02 | 0.09 | 0.09 | 0.02 | 0.07 | 0.15 | 0.02 | 0.07 | 0.13 | 0.02 | 0.09 |
| Somatic | Mean | **158** | **34** | **80** | **317** | **39** | **112** | **256** | **44** | **94** | **174** | **30** | **76** | **127** | **36** | **81** | **167** | **25** | **54** | **154** | **38** | **92** |
|  | SD | 473 | 261 | 461 | 548 | 246 | 634 | 587 | 274 | 430 | 492 | 239 | 447 | 431 | 278 | 479 | 527 | 197 | 317 | 447 | 285 | 513 |
|  | Prop. | 0.26 | 0.06 | 0.10 | 0.56 | 0.08 | 0.11 | 0.47 | 0.08 | 0.13 | 0.26 | 0.05 | 0.09 | 0.22 | 0.06 | 0.10 | 0.29 | 0.05 | 0.08 | 0.24 | 0.07 | 0.11 |
| **Medication** | Mean | **850** | **229** | **349** | **3388** | **128** | **175** | **1078** | **226** | **278** | **795** | **189** | **267** | **845** | **267** | **438** | **789** | **210** | **385** | **877** | **238** | **332** |
|  | SD | 2899 | 3183 | 3783 | 5740 | 867 | 941 | 806 | 3347 | 2847 | 3495 | 2423 | 2327 | 2380 | 3736 | 4869 | 2534 | 2835 | 4347 | 3051 | 3329 | 3497 |
|  | Prop. | 1.00 | 0.63 | 0.72 | 1.00 | 0.64 | 0.68 | 1.00 | 0.61 | 0.69 | 1.00 | 0.65 | 0.74 | 1.00 | 0.61 | 0.71 | 1.00 | 0.50 | 0.60 | 1.00 | 0.68 | 0.77 |
| Psych. | Mean | **42** | **10** | **41** | **28** | **20** | **57** | **37** | **12** | **36** | **36** | **9** | **40** | **50** | **10** | **42** | **49** | **12** | **43** | **40** | **9** | **40** |
|  | SD | 155 | 107 | 206 | 85 | 150 | 269 | 117 | 109 | 177 | 111 | 97 | 213 | 192 | 116 | 203 | 154 | 141 | 271 | 156 | 88 | 168 |
|  | Prop. | 0.24 | 0.06 | 0.21 | 0.11 | 0.04 | 0.11 | 0.22 | 0.04 | 0.15 | 0.24 | 0.06 | 0.22 | 0.24 | 0.06 | 0.22 | 0.26 | 0.05 | 0.18 | 0.23 | 0.06 | 0.23 |
| Somatic | Mean | **807** | **219** | **308** | **3359** | **107** | **118** | **1041** | **214** | **242** | **759** | **180** | **227** | **795** | **257** | **396** | **740** | **198** | **342** | **837** | **229** | **292** |
|  | SD | 2886 | 3180 | 3774 | 5747 | 847 | 897 | 804 | 3344 | 2840 | 3493 | 2420 | 2313 | 2349 | 3733 | 4861 | 2507 | 2829 | 4333 | 3043 | 3326 | 3491 |
|  | Prop. | 1.00 | 0.62 | 0.69 | 1.00 | 0.64 | 0.68 | 1.00 | 0.61 | 0.67 | 1.00 | 0.64 | 0.71 | 1.00 | 0.60 | 0.67 | 1.00 | 0.49 | 0.57 | 1.00 | 0.67 | 0.74 |
| **Resource use** |  |  |  |  |  |  |  |  |  |  |  |  |  |  |  |  |  |  |  |  |  |  |
| **Days in hospital** | Mean | **6.34** | **1.00** | **4.13** | **1.67** | **0.48** | **2.31** | **10.23** | **1.01** | **5.81** | **6.42** | **1.06** | **4.81** | **5.75** | **0.94** | **3.22** | **6.92** | **0.84** | **3.37** | **6.09** | **1.07** | **4.47** |
|  | SD | 18.88 | 7.93 | 18.33 | 5.00 | 5.41 | 13.09 | 27.46 | 9.08 | 23.85 | 19.00 | 8.60 | 19.65 | 17.20 | 7.03 | 15.79 | 24.80 | 8.26 | 16.33 | 15.47 | 7.78 | 19.16 |
|  | Prop. | 0.37 | 0.11 | 0.18 | 0.11 | 0.05 | 0.10 | 0.46 | 0.08 | 0.18 | 0.40 | 0.10 | 0.19 | 0.32 | 0.11 | 0.17 | 0.30 | 0.08 | 0.14 | 0.40 | 0.12 | 0.20 |
| Psych. | Mean | **3.01** | **0.58** | **3.43** | **1.67** | **0.28** | **2.03** | **8.47** | **0.66** | **5.03** | **2.84** | **0.67** | **4.10** | **2.38** | **0.49** | **2.54** | **3.86** | **0.54** | **2.82** | **2.62** | **0.61** | **3.71** |
|  | SD | 15.76 | 7.23 | 17.30 | 5.00 | 4.94 | 12.78 | 27.50 | 8.42 | 22.94 | 13.54 | 8.00 | 18.71 | 15.33 | 6.19 | 14.59 | 19.96 | 7.43 | 15.07 | 13.42 | 7.14 | 18.21 |
|  | Prop. | 0.07 | 0.01 | 0.07 | 0.11 | 0.01 | 0.04 | 0.18 | 0.01 | 0.09 | 0.07 | 0.01 | 0.08 | 0.05 | 0.01 | 0.05 | 0.08 | 0.01 | 0.06 | 0.06 | 0.01 | 0.07 |
| Somatic | Mean | **3.34** | **0.41** | **0.70** | **0.00** | **0.20** | **0.28** | **1.76** | **0.35** | **0.78** | **3.58** | **0.39** | **0.71** | **3.37** | **0.45** | **0.68** | **3.05** | **0.30** | **0.56** | **3.47** | **0.46** | **0.76** |
|  | SD | 10.68 | 2.94 | 4.50 | 0.00 | 2.21 | 2.97 | 3.51 | 3.20 | 5.97 | 13.42 | 2.88 | 3.98 | 8.18 | 2.95 | 4.67 | 15.05 | 3.23 | 4.88 | 7.95 | 2.80 | 4.32 |
|  | Prop. | 0.32 | 0.10 | 0.14 | 0.00 | 0.05 | 0.06 | 0.31 | 0.08 | 0.12 | 0.35 | 0.09 | 0.14 | 0.30 | 0.11 | 0.14 | 0.24 | 0.07 | 0.10 | 0.36 | 0.11 | 0.15 |
| **DDD** | Mean | **619** | **117** | **199** | **333** | **131** | **170** | **438** | **152** | **203** | **554** | **129** | **204** | **709** | **101** | **195** | **877** | **80** | **145** | **502** | **134** | **224** |
|  | SD | 492 | 265 | 385 | 147 | 296 | 396 | 288 | 312 | 346 | 398 | 250 | 357 | 574 | 268 | 416 | 608 | 243 | 360 | 374 | 272 | 393 |
|  | Prop. | 1.00 | 0.62 | 0.72 | 1.00 | 0.64 | 0.68 | 1.00 | 0.60 | 0.68 | 1.00 | 0.64 | 0.73 | 1.00 | 0.60 | 0.70 | 1.00 | 0.50 | 0.60 | 1.00 | 0.68 | 0.77 |
| Psych. | Mean | **72** | **14** | **64** | **21** | **11** | **27** | **55** | **10** | **37** | **73** | **12** | **60** | **75** | **16** | **72** | **82** | **12** | **48** | **68** | **15** | **71** |
|  | SD | 192 | 89 | 199 | 62 | 68 | 107 | 142 | 66 | 126 | 183 | 79 | 184 | 207 | 100 | 221 | 211 | 83 | 182 | 183 | 91 | 206 |
|  | Prop. | 0.24 | 0.06 | 0.21 | 0.11 | 0.04 | 0.11 | 0.22 | 0.04 | 0.15 | 0.24 | 0.06 | 0.22 | 0.24 | 0.06 | 0.22 | 0.26 | 0.05 | 0.18 | 0.23 | 0.06 | 0.23 |
| Somatic | Mean | **546** | **103** | **136** | **312** | **120** | **143** | **383** | **142** | **165** | **481** | **117** | **144** | **634** | **84** | **123** | **796** | **68** | **97** | **434** | **120** | **153** |
|  | SD | 432 | 240 | 293 | 117 | 284 | 373 | 241 | 302 | 312 | 347 | 229 | 269 | 504 | 236 | 310 | 544 | 216 | 274 | 311 | 249 | 300 |
|  | Prop. | 1.00 | 0.61 | 0.68 | 1.00 | 0.63 | 0.66 | 1.00 | 0.59 | 0.65 | 1.00 | 0.63 | 0.70 | 1.00 | 0.59 | 0.66 | 1.00 | 0.48 | 0.55 | 1.00 | 0.67 | 0.73 |
| ***Notes***: Control (1) = balanced for age, birth-assigned sex, and degree of urbanization; Control (2) = balanced for age, birth-assigned sex, degree of urbanization, and psychiatric diagnoses; DDD=defined daily dosed; GD=gender dysphoria; GP=general practitioner; Prop.=proportion of users; Psych.=psychiatric; SD=standard deviation. | | | | | | | | | | | | | | | | | | | | | | |
